# CHOmpact: A reduced metabolic model of Chinese hamster ovary cells with enhanced interpretability

**DOI:** 10.1002/bit.28459

**Published:** 2023-06-05

**Authors:** Ioscani Jiménez del Val, Sarantos Kyriakopoulos, Simone Albrecht, Henning Stockmann, Pauline M. Rudd, Karen M. Polizzi, Cleo Kontoravdi

**Affiliations:** ^1^ School of Chemical & Bioprocess Engineering University College Dublin Dublin Ireland; ^2^ Manufacturing Science and Technology BioMarin Pharmaceutical Cork Ireland Ireland; ^3^ GlycoScience Group National Institute for Bioprocessing Research and Training Dublin Ireland; ^4^ Department of Chemical Engineering Imperial College London London UK; ^5^ Present address: Bioprocessing Technology Institute Agency for Science, Technology and Research (A*STAR) Singapore Singapore; ^6^ Present address: Drug Product Development Janssen Pharmaceuticals Schaffhausen Switzerland

**Keywords:** biopharmaceuticals, Chinese hamster ovary cells, flux balance analysis, nonlinear optimization

## Abstract

Metabolic modeling has emerged as a key tool for the characterization of biopharmaceutical cell culture processes. Metabolic models have also been instrumental in identifying genetic engineering targets and developing feeding strategies that optimize the growth and productivity of Chinese hamster ovary (CHO) cells. Despite their success, metabolic models of CHO cells still present considerable challenges. Genome‐scale metabolic models (GeMs) of CHO cells are very large (>6000 reactions) and are difficult to constrain to yield physiologically consistent flux distributions. The large scale of GeMs also makes the interpretation of their outputs difficult. To address these challenges, we have developed CHOmpact, a reduced metabolic network that encompasses 101 metabolites linked through 144 reactions. Our compact reaction network allows us to deploy robust, nonlinear optimization and ensure that the computed flux distributions are physiologically consistent. Furthermore, our CHOmpact model delivers enhanced interpretability of simulation results and has allowed us to identify the mechanisms governing shifts in the anaplerotic consumption of asparagine and glutamate as well as an important mechanism of ammonia detoxification within mitochondria. CHOmpact, thus, addresses key challenges of large‐scale metabolic models and will serve as a platform to develop dynamic metabolic models for the control and optimization of biopharmaceutical cell culture processes.

## Graphical abstract

CHOmpact presents a reduced metabolic model for CHO cell metabolism and a novel nonlinear objective function. The framework yields intracellular fluxes with increased biological consistency that are easier to analyze and, thereby, provides greater insight into mechanisms governing the physiology of biopharmaceutical production hosts.

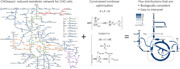

**Abbreviations**
AcCoA: acetyl coenzyme A
Ala: alanine
Aralar1/2: mitochondrial aspartate glutamate carrier 1/2
Arg: arginine
Asn: asparagine
Asp: aspartate
ATP: adenosine triphosphate
Chol: choline
Cit: citrate
CoASH: coenzyme A
DHAP: dihydroxyacetone phosphate
F6P: fructose 6‐phosphate
Form: formate
Fum: fumarate
G6P: glucose 6‐phosphate
GAP: glyceraldehyde 3‐phosphate
Glc: glucose
Gln: glutamine
Glu: glutamate
Gly: glycine
Gly3P: glyceraldehyde 3‐phosphate
Glyc: glycerol
Glyc3P: glycero‐3‐phosphocholine
His: histidine
Ile: isoleucine
Iosbut: isobutyrate
IsobutCoA: isobutyryl coenzyme A
Isoval: isovalerate
IsovalCoA: isovaleryl coenzyme A
Lac: lactate
Leu: leucine
Lys: lysine
Mal: malate
Met: methionine
MTHF: methyltetrahydrofolate
NADH: nicotinamide adenine dinucleotide—reduced
NADPH: nicotinamide adenine dinucleotide phosphate—reduced
NADP^+^
: nicotinamide adenine dinucleotide phosphate—oxidised
NAD^+^
: nicotinamide adenine dinucleotide—oxidised
NH4^+^
: ammonia
OAA: oxaloacetate
OGCP: oxoglutarate/malate carrier protein
Phe: phenylalanine
PhosphoCol: phosphocholine
Pro: proline
PRPP: phosphoribosyl diphosphate
Pyr: pyruvate
q_p_
: cell specific productivity
Ser: serine
Succ: succinate
SuccCoA: succinyl coenzyme A
Symbol: definition
THF: tetrahydrofuran
Thr: threonine
Trp: tryptophan
Tyr: tyrosine
Val: valine
μ_g_
: cell specific growth rate
αKG: alpha ketoglutarate (also oxoglutarate)

## 1 INTRODUCTION

Production of recombinant proteins is known to compete with biomass synthesis for externally provided nutrients. This is particularly true for mammalian cell lines, such as Chinese hamster ovary (CHO) cells, which are the dominant host for the industrial production of therapeutic proteins (O'Flaherty et al., 2020). Metabolic modeling has become an essential tool for understanding resource allocation and, when coupled with advances in genome editing, for designing rational cell engineering strategies. Publication of the CHO‐K1 genome, and the omics analyses this enabled, laid the foundation for systems‐level understanding of this host. This knowledge has been reconstructed mathematically in a community‐curated genome‐scale metabolic model (GeM) of CHO cells termed iCHO1766 (Hefzi et al., 2016). Crucially, iCHO1766 organized knowledge of all biochemical conversions, transport, and exchange reactions to create a large, interlinked network of metabolites and their associated reactions.

The inclusion of gene–protein associations provided a direct link between genes and metabolic reactions. Since then, significant expansions and improvements to iCHO1766 have been achieved, such as gap‐filling studies that also removed dead‐end reactions (Fouladiha et al., 2021), and the integration of a core protein secretory pathway, iCHO2048, enabling the computation of energetic costs and machinery demands of each secreted protein (Gutierrez et al., 2020). iCHO2048 was subsequently used to direct host cell protein knockout studies, which resulted in increased recombinant protein productivity and a cleaner feedstock for downstream purification (Kol et al., 2020), highlighting the power of these models in identifying cellular engineering strategies.

The solution of GeMs, and any underdetermined metabolic model, relies on constraint‐based methods, such as flux balance analysis (FBA), to predict steady‐state intracellular flux distributions (Orth et al., 2010). Although FBA offers the advantage of not requiring detailed knowledge of enzymatic kinetic parameters, it does not return a unique set of intracellular flux values. In addition, the larger the metabolic network considered, the more difficult it becomes to interpret such predictions (Gardner & Boyle, 2017). GeMs, therefore, require large datasets, preferably across different omics levels (e.g., metabolomic, transcriptomic) to increase confidence in results. This is also true for curating GeMs for specific cell lines, raising the need for characterization that goes beyond what is typically conducted in industrial settings.

Several algorithms have been developed to improve the predictive performance of GeMs, for example, by constraining the amount of carbon able to flowthrough reaction fluxes, based on the maximum amount carbon uptake by the cell (ccFBA) (Lularevic et al., 2019), taking into account the selective pressure that exists within cell cultures for fast‐growing cell lines with a low enzyme usage (Lewis et al., 2010), or introducing enzyme capacity constraints (Yeo et al., 2020). Despite these advances, difficulties in handling the models and interpreting their intracellular flux predictions remain unaddressed challenges. An additional limitation is the computational difficulty in creating dynamic versions of GeMs that would reflect the nature of cell culture processes, although recent efforts coupling a CHO GeM with statistical models have yielded promising results in predicting the time evolution of extracellular (EC) amino acid concentrations (Martínez et al., 2015).

In this work, we introduce a reduced‐scale metabolic model, CHOmpact, where the reaction network is based on the work by Carinhas et al. (2013) and has been augmented with a detailed description the aspartate–malate (Asp‐Mal) shuttle, the urea cycle, de novo serine synthesis from glycolytic intermediates, and nucleotide sugar donor (NSD) biosynthesis. The resulting network comprises 101 metabolites and 144 reactions, which due to its compact nature, significantly enhances the interpretability of simulation results. The reduced scale of the reaction network also allows for more complex, nonlinear formulations of the objective function to be incorporated, compared to biomass maximization that is often employed in FBA of GeMs. Our proposed optimization framework allows solution across all phases of cell culture and provides insight into the dynamics of cellular metabolism. We envisage that the advantages presented by CHOmpact will enable the development of dynamic flux balance models that can serve as digital twins for the control and optimization of biopharmaceutical cell culture processes.

## 2 MATERIALS AND METHODS

### 2.1 Cell culture

The GS46 GS‐CHO cell line producing a humanized antitumour‐associated glycoprotein (TAG‐72) IgG4κ mAb (cB72.3), a kind gift by Lonza Biologics, was cultured with three different amino acid feeds: Feed C, Feed U, and Feed U40 (Kyriakopoulos & Kontoravdi, 2014). Triplicate cultures for each feeding regime were performed in orbitally shaken (140 rpm) 250 mL vented conical flasks (Corning) with a 50 mL working volume. The flasks were placed in a humidified incubator with 8% CO_2_ and a temperature of 36.5°C. The basal medium for all cultures was CD CHO (Life Technologies) supplemented with 25 μM methionine sulfoximine (Sigma‐Aldrich). All feeding regimes consisted in adding 10% v/v every 48 h of culture. Feed C used commercial CD EfficientFeed™ C AGT™ (Invitrogen), whereas U and U40 provided amino acids beyond the amounts available in Feed C. Glucose and amino acid concentrations in the feeds are detailed in Kyriakopoulos and Kontoravdi (2014).

### 2.2 Analytical methods

Viable and dead cell density was determined using the trypan blue dye exclusion method. mAb titer was determined using the BLItz® system (Pall ForteBio). Time profiles for glucose, lactate, and ammonia were generated using the Bioprofile 400 analyzer (NOVA Biomedical). Residual amino acid profiles were quantified with the PicoTag method (Waters) on an Alliance HPLC instrument (Waters). EC pyruvate concentrations were determined with an enzyme assay kit (Abcam). mAb Fc glycoprofiling was performed with an automated sample preparation workflow (Stockmann et al., 2013) where mAb samples were affinity‐purified from the cell culture supernatant with a 96‐well protein G immunoglobulin G purification plate (Thermo Fisher Scientific). Glycans were released from the mAb through PNGase (Prozyme) digestion and labeled with 2‐amino benzamide (Ludger). Labeled glycans were separated using hydrophilic interaction ultra performance liquid chromatography and quantified with fluorescence detection (Stöckmann et al., 2013). Glycans were initially assigned by comparing their Glucose Unit retention times with those available in the national institute for bioprocessing research & training GlycoBase 3.2 structural *N*‐glycan library (Campbell et al., 2008). Glycan assignment was confirmed through weak anion exchange chromatography and quadrupole time‐of‐flight mass spectrometry on exoglycosidase‐digested samples (Albrecht et al., 2014).

### 2.3 Dry cell weight measurement

Duplicate cultures were harvested at Days 4 (mid‐exponential) and 10 (stationary) for dry cell weight measurements. First, viable cell density was determined using trypan blue dye exclusion. Immediately after cell counting, 40 mL of the cultures was harvested and centrifuged at 1000*g* for 1 min in preweighed 50 mL falcon tubes. After discarding the supernatant, cell pellets were washed once with 40 mL 0.9% w/v NaCl (Sigma‐Aldrich) and centrifuged at 1000*g* for 1 min. The wash was discarded, and the cell pellet was left to dry in a nonhumidified incubator at 37°C until no changes in weight were observed. Tubes were weighed within 1 mg accuracy (ACCULAB; Sartorius).

### 2.4 Data processing and analysis

The cell‐specific rates for nutrient consumption and metabolite/product secretion, $q_{i}\left(t_{n}\right)$, were calculated with Equation 1 (Sauer et al., 2000), where $N_{i,\mathrm{cons}}\left(t_{n}\right)$ is the consumed/produced amount of component i up to time $t_{n}$ and $\mathrm{IVC}\left(t_{n}\right)$ is the integral of viable cells up to time $t_{n}$.

(1) $$N_{i,cons}\left(t_{n}\right)=q_{i}\left(t_{n}\right)IVC\left(t_{n}\right),$$

(2) $$N_{i,cons}\left(t_{n}\right)={\left[C\right]}_{i,res}\left(t_{n}\right)Vol\left(t_{n}\right)-\sum_{j=1}^{n}{\left[C\right]}_{i,feed}Vol_{fed}\left(t_{j}\right),$$

(3) $$IVC\left(t_{n}\right)=\sum_{j=1}^{n}\frac{\left[X_{v}\right]\left(t_{j}\right)Vol\left(t_{j}\right)+\left[X_{v}\right]\left(t_{j-1}\right)Vol\left(t_{j-1}\right)}{2}\left(t_{j}-t_{j-1}\right),$$

(4) $$f_{k}=\frac{{\left[mAb\right]}_{k}\left(t_{j}\right)-{\left[mAb\right]}_{k}\left(t_{j-1}\right)}{\left[mAb\right]\left(t_{j}\right)-\left[mAb\right]\left(t_{j-1}\right)}.$$

$N_{i,\mathrm{cons}}\left(t_{n}\right)$ and $\mathrm{IVC}\left(t_{n}\right)$ were computed using Equations 2 and 3, where ${\left[C\right]}_{i,\mathrm{res}}\left(t_{n}\right)$ is the residual concentration of component i at time $t_{n}$, ${\left[C\right]}_{i,\mathrm{feed}}$ is the concentration of component i in the feed, ${\mathrm{Vol}}_{\mathrm{fed}}\left(t_{j}\right)$ is the feed volume added at time j, $\left[X_{v}\right]\left(t_{j}\right)$ is cell density at time $t_{j}$, and $\mathrm{Vol}\left(t_{j}\right)$ is liquid volume in the culture flask at time $t_{j}$. Linear regressions to obtain $q_{i}\left(t_{n}\right)$ were performed with the LINEST function in Microsoft Excel. Confidence intervals for the obtained values were computed with the least square residuals and a *t* value for p=0.05. Five intervals with constant uptake/secretion rates were identified: early exponential, mid‐exponential, late exponential, early stationary, and stationary. Supporting Information: Figures 1 and 2 present raw and processed culture data, respectively. $f_{k}$, the fraction of mAb glycoform secreted from time $t_{n-1}$ to $t_{n}$, was calculated with Equation 4, where ${\left[\mathrm{mAb}\right]}_{k}\left(t_{n}\right)$ is the concentration of mAb glycoform k present at time $t_{n}$ and $\left[\mathrm{mAb}\right]\left(t_{n}\right)$ is the total mAb titer at time $t_{n}$ (Fan et al., 2015; del Val, Fan, et al., 2016;).

### 2.5 Flux balance model development

**Figure 1:**
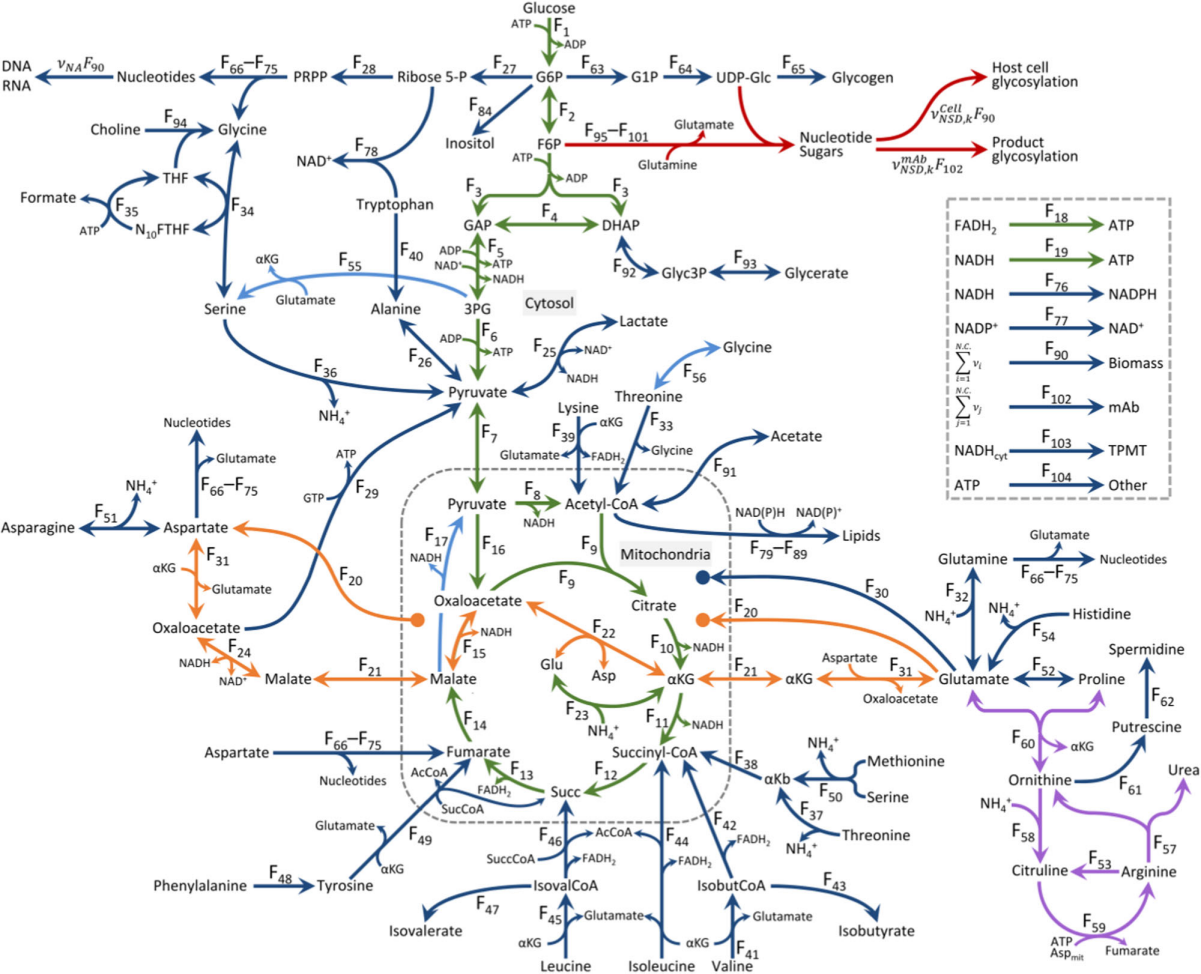
CHOmpact reaction network CHOmpact considers 101 species linked through 144 fluxes. Different colors indicate particular metabolic pathways: glycolysis, tricarboxylic acid, and oxidative phosphorylation (green), nucleotide sugar donor metabolism (red), aspartate–malate shuttle (orange), urea cycle (purple), amino acid and nucleotide metabolism (dark blue), and cycle fluxes that must be constrained and/or estimated during optimization (light blue).

The CHOmpact flux balance model (Figure 1) is based on previous work by Carinhas et al. (2013) and has been expanded to include the Asp‐Mal shuttle (Mulukutla et al., 2012; Nolan & Lee, 2011), the urea cycle (Zamorano et al., 2010), de novo serine synthesis from glycolytic intermediates, and NSD biosynthesis (Kremkow & Lee, 2018). Details on the Asp‐Mal shuttle were included with the aim of gaining further insight into glutamate and aspartate anaplerosis and cataplerosis. Reactions of the urea cycle, catalyzed by enzymes whose genes are present in CHO cells, were added to provide additional avenues for ammonia detoxification and better insight into arginine metabolism. De novo serine synthesis was included to ensure that this prototrophic amino acid in CHO cells is not growth limiting. NSD biosynthesis was included to gain insight into the metabolic burden of cellular and recombinant product glycosylation on CHO cell metabolism.

Manual curation of our FBA model was performed using the kyoto encyclopedia of genes and genomes (KEGG) database (Kanehisa et al., 2017, 2019) and the reference CHO‐K1 and *Cricetulus griseus* genome annotations (Kremkow et al., 2015; Lewis et al., 2013; Rupp et al., 2018). Sequential reactions were combined into single reaction fluxes to reduce degrees of freedom within the model (Nolan & Lee, 2011). CHOmpact is comprised of material balances for 101 species, an additional equation that defines the consumption of ATP toward active amino acid transport, and 144 fluxes (Figure 1 and Supporting Information: Table 1) yielding 42 degrees of freedom. Supporting Information: Table 1 provides links for all enzymatic reactions in KEGG (Kanehisa et al., 2017, 2019) and to reference sequences in the NCBI database (O'Leary et al., 2016). Supporting Information: Table 2 presents the stoichiometric matrix of CHOmpact.

### 2.6 Stoichiometric equations for biomass and product

Calculations for the biomass stoichiometric coefficients are presented in Supporting Information: Table 3. Based on our measurements, the biomass equation considers a dry cell weight of 219 pg/cell for exponentially growing cells and 311 pg/cell for cells in the stationary phase. The mass composition of GS‐CHO cells is based on Sheikh et al. (2005) and Hefzi et al. (2016) and assumes 74.2% protein, 11.1% lipids, 5.0% RNA, 1.4% DNA, 0.4% glycogen, 0.2% *N*‐glycans, 0.3% *O*‐glycans, 2.9% other intracellular components (e.g., methyltetrahydrofolate, nicotinamide adenine dinucleotide phosphate—reduced, acetyl coenzyme A) and 4.5% nonbalanced components.

The amino acid composition of cellular proteins was computed from CHO cell proteomic data (Baycin‐Hizal et al., 2012), as reported by del Val, Polizzi, et al. (2016). The glycan content of biomass has been included by using stoichiometric coefficients for cellular glycolipid and *N*‐ and *O*‐linked protein glycosylation (del Val, Polizzi, et al., 2016). The stoichiometric equation for the cB72.3 humanized IgG4κ mAb was computed using the amino acid sequences for human IgG4 Fc (Heilig et al., 2003), the human kappa light chain constant fragment (Brady et al., 1991), and the variable heavy and light chain fragments for cB72.3 (Xiang et al., 1999), as shown in Supporting Information: Table 4.

mAb glycoprofiling at three culture timepoints (192, 240, and 288 h) allowed us to calculate stoichiometric coefficients for NSD consumption toward mAb glycosylation across three culture intervals: 0–192 , 192–240 , and 240–288 h. These calculations were made with Equation 4, and the obtained stoichiometric values are presented in Supporting Information: Table 5.

### 2.7 FBA solution: Nonlinear optimisation

As with most FBA models, no intracellular accumulation of species has been assumed in the material balances generated from our stoichiometric matrix, leading to a problem of the form S×F=0, where S is the stoichiometric coefficient matrix presented in Supporting Information: Table 2 and F is the vector of unknown fluxes. Because the model contains more unknown fluxes (144) than equations (102), it is solved using constraint‐based optimization (Banga, 2008).

Two constraint‐based optimization strategies were used to solve CHOmpact (Table 1). The first maximizes the rate of biomass synthesis while maintaining the transport flux for all nutrients, metabolites, and product set to experimentally determined values. Supporting Information: Table 6 presents the experimental data used for optimization. Reaction reversibility constraints, based on enzyme data available in KEGG (Kanehisa et al., 2017, 2019) and BRENDA (Jeske et al., 2019), were included as indicated in Supporting Information: Tables 1 and 2.

**Table 1:** Optimization strategies for flux balance model solution.

BM maximization	Nonlinear optimization
S×F=0	S×F=0
$\mathrm{MAX}\left(F_{142}\right)$	$\mathrm{MIN}\left(\frac{\sum_{j=1}^{104}F_{j}^{2}}{ATP_{\mathrm{synth}.}}+SSE+\overset{14}{\underset{k=1}{\sum }}F_{k,BP}^{2}+\frac{F_{17}}{F_{14}}\right)$
Subject to	Subject to
$\left\{\begin{array}{l} LB_{i}\leq F_{i}\leq UB_{i}\\ {\left[100\left(\frac{F_{143}}{q_{p}}-1\right)\right]}^{2}\leq \varepsilon_{p} \end{array}\right..$	$\left\{\begin{array}{l} LB_{i}\leq F_{i}\leq UB_{i}\\ 0\leq \frac{F_{17}}{F_{14}}\leq 1\\ C_{Asp/Mal}\leq \varepsilon_{Asp/Mal}\\ {\left[100\left(\frac{F_{16}}{F_{105}}-0.01\right)\right]}^{2}\leq \varepsilon_{16/105} \end{array}\right.,$
	where
	$ATP_{\mathrm{synth}.}={\left(F_{5}\right)}^{2}+{\left(F_{6}\right)}^{2}+{\left(1.5F_{18}\right)}^{2}+{\left(2.5F_{19}\right)}^{2},$
	$SSE=\overset{38}{\underset{k=1}{\sum }}{\left[100\left(\frac{F_{m}^{Comp.}}{F_{m}^{Meas.}}-1\right)\right]}^{2},$
	$C_{Asp/Mal}={\left(\overset{18}{\underset{g}{\sum }}\nu_{g}F_{g}-F_{20}\right)}^{2}.$

Abbreviations: SSE, sum of square error.

The optimization strategy proposed herein simultaneously maximizes the fluxes where ATP is synthesized, while minimizing the sum of squared intracellular fluxes. This objective function represents maximum energetic efficiency by the cells (Schuetz et al., 2007) and was used to ensure consistent directionality of central carbon metabolism fluxes. Alongside maximizing the energetic efficiency of the cells, the sum of squared differences between measured and computed fluxes was minimized to ensure consistency between model results and experimental measurements. To avoid flux F_15_ (Mal_mit_ + NAD^+^ ↔ OAA_mit_ + NADH_mit_) being bypassed by F_17_ (Mal_mit_ + NAD^+^ → Pyr_mit_ + CO_2_ + NADH_mit_), the F_17_/F_14_ ratio was constrained to values within [0, 1] and minimized. If this constraint is not present, F_15_, may be reversed and possibly lead to stalling the tricarboxylic acid (TCA) cycle by the absence of carbon flowing through F_9_ (AcCoA + OAA_mit_ → Cit + CoASH). The secretion fluxes of nonmeasured by‐products (except CO_2_) were minimized, and flux reversibility constraints were also included within the CHOmpact optimization strategy. Our rationale for minimizing the secretion of nonmeasured fluxes intends to ensure that most of the carbon is destined toward energy production (correct TCA directionality) and that amino acids are mainly consumed toward biomass synthesis. It is worth noting that the growth rate maximization strategy that is prevalently used to solve flux balance models also implies by‐product secretion minimization (i.e., it pushes nutrients toward biomass synthesis). Although we acknowledge that CHO cells are reported to secrete a multitude of only recently compiled metabolites (Pereira et al., 2018), we believe that including this term in the objective function is valid and consistent with more traditional FBA objective functions.

The maximum rate with which Glu is transported into mitochondria (F_20_, via the mitochondrial aspartate glutamate carrier 1/2 (Aralar1/2) Glu/Asp antiporters) was constrained to be the sum of fluxes where it is produced in reactions that do not belong to the Asp‐Mal shuttle. F_20_ was constrained to limit the magnitude of fluxes through the Asp‐Mal shuttle because, if left unconstrained, they can take any value as long as the cytosolic and mitochondrial NADH fluxes are balanced (see Supplementary File). F_20_ is the most appropriate target for constraint because it is the rate‐limiting step of the Asp‐Mal shuttle (LaNoue et al., 1974; LaNoue & Tischler, 1974). F_16_ was constrained to be 1% of the glucose uptake flux (F_105_), as previously measured for CHO cells (Ahn & Antoniewicz, 2013).

Both optimization strategies are outlined in Table 1, where $F_{j}$ are all intracellular fluxes, ${\mathrm{ATP}}_{\mathrm{synth}.}$ is the squared sum of ATP synthesis reaction fluxes, SSE is the sum of square errors (SSEs) between the measured and computed transport fluxes, $F_{k,\mathrm{BP}}$ are the fluxes of by‐product synthesis reactions, ${\mathrm{LB}}_{i}$ and ${\mathrm{UB}}_{i}$ are lower and upper bounds for flux values (a reaction is irreversible when ${\mathrm{LB}}_{i}=0$), $C_{\mathrm{Asp}/\mathrm{Mal}}$ is the Asp‐Mal shuttle constraint, ε represents a small threshold value, $F_{m}^{\mathrm{Comp}.}$ and $F_{m}^{\mathrm{Meas}.}$ are the computed and measured transport fluxes, $F_{g}$ are the fluxes where Glu is produced or consumed (excl. Asp‐Mal shuttle), and $\nu_{g}$ is the stoichiometric coefficient for the Glu reactions. The final constraint in the BM maximization ensures that the difference between the experimental ($q_{p}$) and computed ($F_{143}$) specific productivity is below a small threshold value ($\varepsilon_{p}$).

The biomass maximization strategy was used to compare the performance of CHOmpact with the iCHO1766 GeM (Hefzi et al., 2016), while energetic efficiency maximization was used for all other simulations. All optimizations were performed using the nonlinear programming sequential quadratic programming solver (NLPSQP) of gPROMS ModelBuilder v7.0.9 (Process Systems Enterprise, 2021) on a standard workstation (AMD Ryzen 3600X @ 4.2 GHz and 32 GB RAM).

## 3 RESULTS AND DISCUSSION

### 3.1 CHOmpact performs comparably with the iCHO1766 GeM

The specific growth rates ($\mu_{g}$) of multiple CHO cell lines cultured under different conditions were calculated through the growth rate maximization strategy. The inputs for this solution strategy, namely the nutrient, metabolite, and product transport fluxes, were obtained from previously published data (Carinhas et al., 2013; Martínez et al., 2015; Selvarasu et al., 2012), as summarized in the Supporting Information of Hefzi et al. (2016).

**Figure 2:**
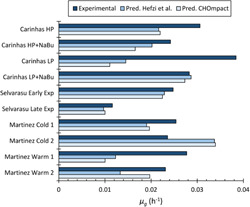
Comparison of experimentally determined and predicted specific growth rates (*μ_g_*) for different Chinese hamster ovary cell lines and culture conditions. The dark blue bars present the experimentally determined *μ_g_* values reported by Carinhas et al. (2013), Martínez et al. (2015), and Selvarasu et al. (2012). The medium blue bars present predictions reported by Hefzi et al. (2016), and the light blue bars show the *μ_g_* values predicted with CHOmpact.

Figure 2 compares our predicted $\mu_{g}$ values with those obtained with iCHO1766 to assess whether our assumed biomass composition and reduced reaction network performs comparably with the consensus GeM. CHOmpact predicts the $\mu_{g}$ for Car LP + NaBu, Selv Early Exp, and Selv Late Exp within 15% deviation. CHOmpact underpredicts the growth rate of Car HP, Car HP + NaBu, Mart Warm 1, Mart Warm 2, and Mart Cold 1, while it overpredicts the $\mu_{g}$ of Mart Cold 2.

$\mu_{g}$ optimizations were performed on CHOmpact using the biomass composition assumed by Hefzi et al. (2016) to discern whether it was the cause for deviations in predictive capabilities. These optimizations yielded deviations from the experimental data that are indistinguishable from those obtained with our assumed biomass composition (data not shown). These results are expected when considering that the differences between our assumed biomass composition and the one used in the GeM involve prototrophic amino acids only (Ala, Gln, Gly) (Supporting Information: Figure 3).

Although deviations are observed, our predictions are similar to those reported for the iCHO1766 GeM and are better for Car HP, Selv Late Exp, Mart Cold 1, and Mart Warm 2. Overall, iCHO1766 has an average percent deviation across the 10 experimental $\mu_{g}$ values of 30%, whereas CHOmpact has an average deviation of 30.3%. When considering that our model includes only 144 reactions when compared to 6663 in iCHO1766, a marginal reduction in predictive capability is acceptable, especially considering gains in model output consistency, interpretability, and reductions in computational expense when using nonlinear optimization.

### 3.2 CHOmpact identifies the source of *μ_g_* prediction inaccuracies

Our proposed nonlinear optimization strategy enables the identification of nutrient uptake rates that lead to differences between calculated and experimental‐specific growth rates. This is achieved by fixing the biomass growth rate and the uptake/secretion rates of Glc, Lac, NH_4_^+^, Pyr, Ala, Asn, Asp, Glu, Gly, and product to the experimentally determined values. The uptake fluxes of the remaining (mainly auxotrophic) amino acids are obtained via constrained optimization, where the SSE between the experiment and measurement is minimized. This strategy ensures that the solution matches the experimental growth rate and specific productivity while finding the combination of amino acid uptake fluxes that minimizes deviations from the experimental values.

The above strategy yields the results presented in Table 2, where the percent increases in specific uptake rates required to match the experimental values of $\mu_{g}$ and $q_{p}$ are shown (positive/red values denote the percent increase in uptake fluxes required to match $\mu_{g}$ and $q_{p}$). Table 2 shows that, for Car LP + NaBu, Selv Early Exp, Selv Late Exp, and Mart Cold 2, small or no increases in amino acid uptake rates are required to match $\mu_{g}$ and $q_{p}$. This is expected, considering the results of Figure 2, where the predicted values for $\mu_{g}$ are matched or exceeded for these datasets.

**Table 2:** Percent increases in amino acid uptake rates required to match *μ*_g_ and *q*_*p*_.

	Car HP (%)	Car HP (NaBu) (%)	Car LP (%)	Car LP (NaBu) (%)	Selv (Early) (%)	Selv (Late) (%)	Mart (Cold 1) (%)	Mart (Cold2) (%)	Mart (Warm 1) (%)	Mart (Warm 2) (%)
*q*_*Arg*_	0.0	0.0	8.5	−0.1	−0.3	0.0	1.0	−0.8	17.3	14.3
*q*_*Gln*_	0.0	0.0	0.0	0.0	0.0	0.0	0.0	0.0	48.5	36.5
*q*_*His*_	0.0	25.0	33.9	0.0	0.0	2.6	1.8	2.8	22.7	7.9
*q*_*Ile*_	0.0	0.0	19.8	0.0	0.0	0.0	2.7	3.4	9.5	10.5
*q*_*Leu*_	12.6	1.4	29.6	0.0	0.4	9.0	8.6	2.8	18.6	15.0
*q*_*Lys*_	24.7	0.0	38.1	0.0	0.0	4.0	22.0	4.1	41.0	16.7
*q*_*Met*_	9.4	0.0	23.4	0.0	0.0	0.0	−0.2	0.1	13.8	0.0
*q*_*Phe*_	25.4	0.0	47.4	3.3	8.4	10.1	16.4	0.9	33.2	6.1
*q*_*Pro*_	0.0	0.0	21.4	0.0	0.0	0.0	3.1	1.3	2.6	6.3
*q*_*Ser*_	0.1	0.1	41.8	0.1	0.0	0.0	9.3	6.7	26.4	34.0
*q*_*Thr*_	2.8	0.0	15.2	0.0	0.6	0.1	6.5	6.7	9.8	14.0
*q*_*Trp*_	0.0	0.0	6.0	0.0	0.0	0.0	0.8	0.5	20.0	2.1
*q*_*Tyr*_	21.8	0.0	41.7	2.6	0.0	0.0	5.5	0.4	28.3	4.6
*q*_*Val*_	12.4	5.1	34.1	0.0	0.6	6.8	3.6	2.5	17.1	12.2

*Note*: The intensity of the colors (from green = 0% to red) corresponds to magnitudes across all datasets.

Across all other datasets, substantial increases in amino acid uptake rates are required to match the experimental values for $\mu_{g}$ and $q_{p}$. Except for both Mart Warm datasets, the amino acids which limit growth are auxotrophic (shown in Table 2) and, in all but one case (Car HP NaBu), multiple auxotrophic amino acids limit growth rate. Sharp deviations across multiple amino acids are observed for the Car LP and both Mart Warm datasets.

Underestimations for $\mu_{g}$ arise from two possible sources: (i) either the assumed stoichiometric coefficients for auxotrophic amino acids in biomass are too high or (ii) the measured uptake rates for auxotrophic amino acids are underestimated. Errors in uptake rate measurements above 30% are unlikely (see Table 2); therefore, uncertainty in biomass weight and composition are likely responsible, especially when considering they are seldom measured for flux balance studies.

Széliová et al. (2020) recently reported the average protein content and dry cell weight of multiple CHO cell lines cultured under different conditions as 55.7 ± 5.5% w/w and 262.1 ± 28.2 pg/cell, respectively. When comparing these values with the 74.2% w/w protein and 350 pg/cell assumed for the datasets with the largest underprediction in $\mu_{g}$ (Mart Warm), a maximum reduction of 45.3% in amino acid demand toward biomass synthesis can be computed. This reduction compensates for the calculated shortfalls in amino acid uptake rates presented in Table 2 and would improve the predictive capability of CHOmpact and Hefzi's GeM.

The results of Figure 2 and Table 2 were generated using values of 350 pg/cell and 74.2% w/w protein to pinpoint how model predictive capability is impacted by the reduced reaction network when compared with the full GeM. All subsequent calculations herein are performed using our measured DCW (219 pg/cell for exponential growth and 311 pg/cell for stationary phase) and a protein content of 74.2% because the corresponding stoichiometric coefficients are similar to those obtained with data from Széliová et al. (2020) (Supporting Information: Figure 4), despite considerable differences with their measured DCW (262.1 ± 28.2 pg/cell) and protein content (55.7 ± 5.5%).

These results highlight the importance of using accurate measurements for DCW and biomass composition in flux balance modeling. Biomass composition will impact growth rate predictions only when the uptake of auxotrophic constituents of biomass and product are limiting. The opposite occurs for the Mart Cold 2 data set, where both the GeM and CHOmpact overpredict $\mu_{g}$.

Several strategies have been developed to address situations where cell growth is not limited by stoichiometry. Different metabolic objectives, such as maximizing energetic efficiency or minimizing redox stress (Chen et al., 2019; Feist & Palsson, 2010; Schuetz et al., 2007), can improve the predictive capability of flux models. Alternatively, strategies to further constrain fluxes through the reaction network can be deployed. Lularevic et al. (2019) have constrained fluxes using carbon balancing, and Yeo et al. (2020) have combined the maximum reaction rates and expression levels of metabolic enzymes to constrain fluxes through the metabolic network.

### 3.3 The CHOmpact objective function is robust and enhances the biological consistency of intracellular flux distributions

#### 3.3.1 CHOmpact objective function robustness

The CHOmpact objective function is nonlinear, and when using a local nonlinear solver, such as gPROMS NLPSQP, there is uncertainty on which local optimum is being obtained and whether its value is indeed the optimal one across the search space. Importantly, the solution obtained through nonlinear optimization is often contingent on the initial values (guesses) of the decision variables (Zhang & Boley, 2022).

To assess the consistency and robustness with which our proposed objective function finds the optimum point across the search space, 81 (3^4^) optimizations were run with combinations of three initial guesses—low, medium, and high (L/M/H)—for four key decision variables, F_77_, F_103_, F_104_, and F_106_, using input data for the Feed C Early Exp culture interval. All other optimization decision variables were excluded from this analysis because they correspond to transport fluxes to/from the cells whose initial guesses were always set to experimentally determined values. The L/M/H values were set to the lower bound, the midpoint, and the upper bound for each decision variable. The Feed C Early Exp culture interval was selected because it was observed to have the highest objective function value and the lowest convergence rate.

Of the 81 optimizations, 25 failed because they did not meet NLPSQP convergence criteria, while 30 of the successfully converged optimizations reached the same minimum value of 552.8. The remaining 26 calculations identified secondary local minima between the [552.29, 586.12] and [713.64, 810.52] ranges. Although this analysis confirms the influence initial guesses have on the obtained local optimum, most of the initial value sets tested reach the optimal objective function value. Our analysis also identified the subset of initial value combinations that are more likely to reach the optimum point. Based on the above, all optimizations performed hereafter were run in triplicate (with three sets of initial values from the identified subset) to ensure that the local optimum is robustly identified. Detailed results for this analysis are presented in Supporting Information: Figure 5.

#### 3.3.2 CHOmpact objective function versus SSE

To highlight how the additional terms of the CHOmpact objective function (energy efficiency, by‐product, and F_17_/F_14_) contribute to the biological consistency of the obtained intracellular flux distributions, we compared its results with those obtained through SSE minimization, the most widely used strategy in metabolic flux analysis. Results for this analysis are presented in Supporting Information: Figure 6.

Only small differences between CHOmpact and SSE minimization were observed for glycolytic and pentose phosphate pathway reactions. However, considerable differences can be seen across the Asp‐Mal shuttle fluxes (F_20_–F_24_), where SSE obtains much higher flux values for all three feed datasets. For example, SSE minimization obtains F_20_ (the Asp/Mal shuttle Aralar1/Aralar2 antiport transport of Glu/Asp into/from mitochondria) flux values of 82.2, 75.5, and 61.8 nmol/10^6^ cells/h across the Feed C Mid Expon., Late Expon., and Early Stat. intervals, respectively. For the same input datasets, the CHOmpact objective function obtains values of 23.7, 6.5, and 13.4 nmol/10^6^ cells/h, respectively. Across those culture intervals, Glu uptake from the culture media does not exceed 1.3 nmol/10^6^ cells/h. This potential inconsistency obtained with the SSE objective function likely arises from the cyclical nature of the Asp‐Mal shuttle, where all but the NAD+/NADH fluxes cancel out (as outlined in the Supplementary File). The additional terms of the CHOmpact objective function (and associated constraints—e.g., the Asp‐Mal constraint) were included precisely to address such concerns and yields more biologically consistent intracellular flux distributions.

### 3.4 CHOmpact facilitates easier interpretation of flux distributions

Our optimization strategy provides enhanced insight into metabolic dynamics by enabling the calculation of flux distributions during cell culture phases beyond exponential growth. In addition, our reduced reaction network simplifies model output interpretation to better relate the obtained flux distributions with cellular physiology.

#### 3.4.1 Flux distribution dynamics

**Figure 3:**
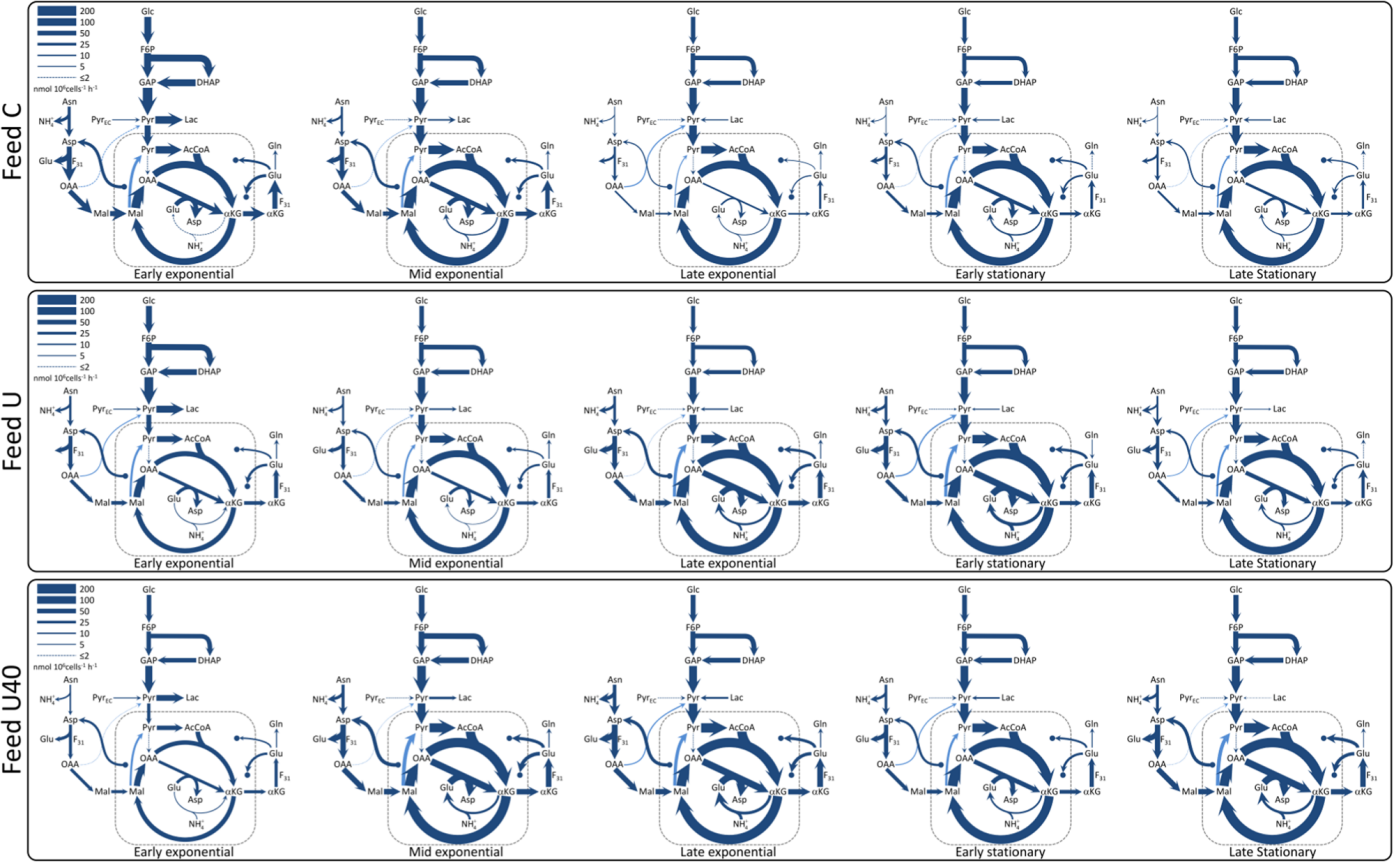
Flux map comparison. The flux distributions for central carbon metabolism and the aspartate/malate shuttle are shown for five culture intervals (early, mid, and late exponential as well as early and late stationary) across three feed compositions (Feed C, U, and U40). The line thickness corresponds to flux magnitude as indicated by the legend at the top left‐hand corner of each box.

Figure 3 presents central carbon metabolism and Asp‐Mal shuttle fluxes obtained through nonlinear optimization on CHOmpact, where the flux maps correspond to three different feed compositions (Feed C, Feed U, and Feed U40) across five phases of culture. For all feed compositions, the glycolytic fluxes decrease with time, with the highest fluxes observed during early exponential growth and the lowest during the stationary phase. The glycolytic flux magnitude is largely determined by the glucose uptake rates, which steadily decrease as the culture progresses (Supporting Information: Figure 2). Conversely, fluxes through the TCA pathway increase with culture time, which occurs because reduced lactate production allows for more glycolysis‐derived pyruvate to reach mitochondria. The high rates of glycolysis and lactate production observed during the initial growth phases of culture, often referred to as the Warburg effect (Vander Heiden et al., 2009), have been widely reported in CHO cells (Buchsteiner et al., 2018; Kelly et al., 2018).

The calculated fluxes through the Asp‐Mal shuttle are defined by our imposed constraint on F_20_, which limits its value to the flux of “available” Glu (i.e., the sum of fluxes where this amino acid is produced that are not involved in the Asp‐Mal shuttle). If left unconstrained, Asp‐Mal shuttle fluxes have been reported to reach values that are comparable to those through central carbon metabolism (Mulukutla et al., 2012; Nolan & Lee, 2011) (also see Supporting Information: Figure 6). Although mathematically correct, these excessive flux values would be limited by cytosolic Glu availability, which is the rate‐limiting substrate for the Asp‐Mal shuttle (LaNoue et al., 1974; LaNoue & Tischler, 1974). Despite not directly representing Glu availability, our proposed constraint makes the Asp‐Mal shuttle fluxes fall below those of glycolysis and, thereby, makes them more biologically consistent.

#### 3.4.2 Glutamate anaplerosis and cataplerosis

**Figure 4:**
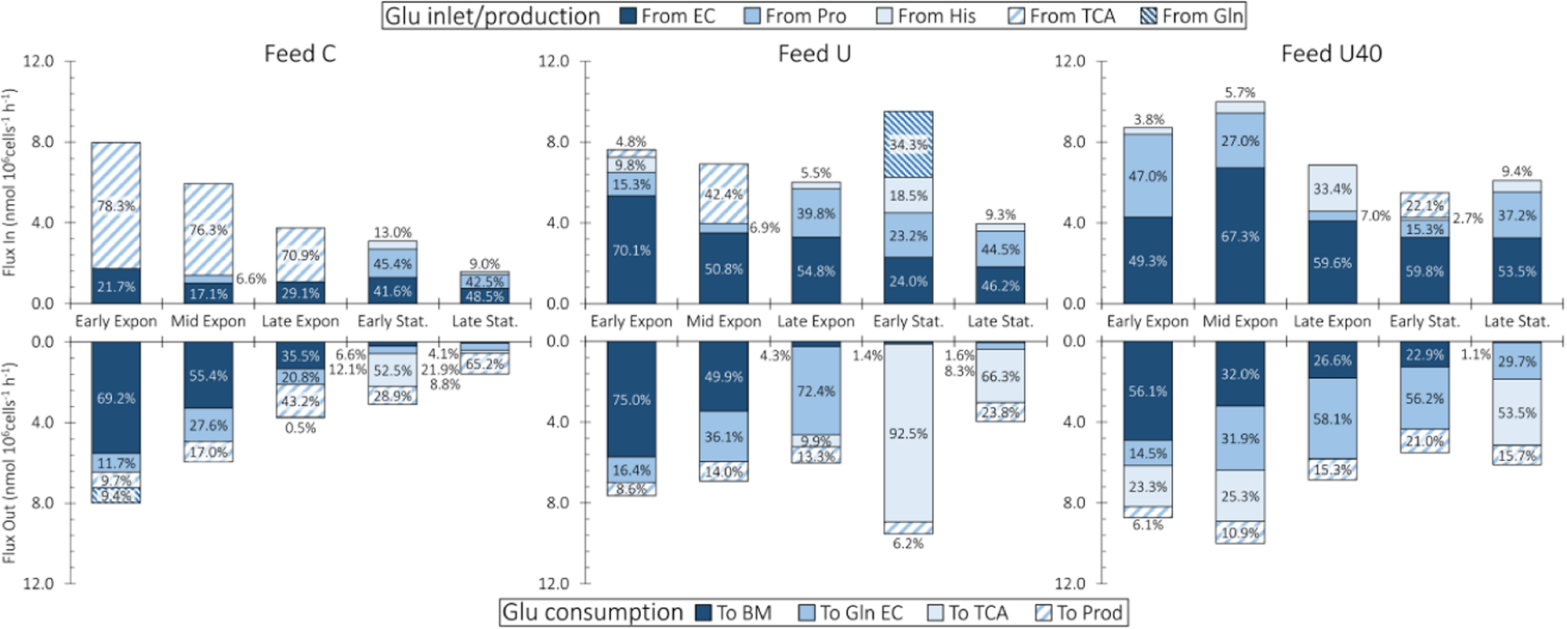
Glutamate flux distributions. The glutamate inlets for all feed compositions are shown in the top half and the outlets are shown in the bottom half. Glutamate sources and sinks are indicated by the shading and the top and bottom legends. The percent contributions to the total inlet or outlet are shown within the bars.

Glutamate can either be consumed toward TCA and energy production (anaplerosis) or produced from TCA metabolites for subsequent use in biomass synthesis (cataplerosis). In the context of our flux balance model, Glu anaplerosis is observed when more of this amino acid is transported into mitochondria (F_20_ + F_30_) than what is transported out, in the form of αKG. Net Glu cataplerosis is observed when less of it is transported into mitochondria than the αKG transported out. Importantly, however, anaplerosis and cataplerosis are driven by the balance of species along the TCA cycle, not solely amino acid metabolism. Glu transport into/out of mitochondria is a consequence of carbon shortage/excess within the TCA (Owen et al., 2002). Figure 4 shows that, in Feed C and Feed U, net Glu cataplerosis is observed during the exponential growth intervals and is reversed during stationary phase, when net anaplerosis occurs. Feed U40 contrast with Feed C and Feed U by presenting Glu anaplerosis during early phases of culture (Figure 4—bottom).

Feed U presents considerable anaplerosis during the early stationary interval, where it accounts for 92.5% of total consumed Glu (Figure 4—bottom). This high anaplerosis is associated with increased Glu production from Gln (37.3% of total) and, to a lesser extent, from His (18.5% of total) (Figure 4—top). Interestingly, this is the only culture condition and interval where Gln is consumed from the extracellular environment (Supporting Information: Figure 1). In Feed U40, Glu cataplerosis is only observed during the early stationary interval, where 21.4% of all Glu produced is derived from TCA (Figure 4—top). Glu cataplerosis during early stationary phase most likely arises to offset the high level of Gln secretion observed during this interval (Supporting Information: Figure 1).

In Feed C, Glu cataplerosis is driven by the high Asn uptake rates observed during exponential growth, where F_31_ provides a path for Asn overflow toward pyruvate via oxaloacetate (F_29_) and malate (F_24_ and F_21_). An alternative cataplerotic pathway for Glu would be its direct synthesis from cytosolic αKG via a cytosol‐localized EC 1.4.1.3 enzyme. Such an enzyme would consume NH_4_^+^ and produce cytosolic NAD^+^ (Yang et al., 2005) and could, thereby, reduce lactate production (Freund & Croughan, 2018). These properties would make a cytosolic version of EC 1.4.1.3 an interesting target for metabolic engineering; however, the endogenous version of this enzyme is not localized in the cytosol of CHO cells.

Prior flux balance work on standard (non‐GS) CHO cells commonly reports net Glu anaplerosis during the exponential growth phase of cells, where high lactate production results in low TCA fluxes (Ahn & Antoniewicz, 2013). Glu anaplerosis is thought to be used by cells to replenish flux through TCA, and is also known to be a major source of ammonia production because much of this anaplerosis involves glutamine (Gln) deamidation (Dean & Reddy, 2013; Wahrheit et al., 2014).

In contrast to past work, our FBA results indicate substantial Glu anaplerosis during the stationary phases of culture (Figure 4—bottom), and that this anaplerosis arises from the high uptake rate of Asn observed across all cultures. The Asn overflow pathway produces Glu in F_31_ which, in turn, causes Glu overflow that is taken up by TCA. Anaplerosis as a means to cope with Glu overflow is also substantiated by the early stationary phase flux distribution of Feed U, which presents the highest level of Glu anaplerosis observed across all datasets (8.81 nmol/10^6^ cells/h) (Figure 4—bottom). This culture interval is the only one where Gln is consumed to produce Glu through deamidation, as is often reported for non‐GS‐CHO cells.

Glu anaplerosis is commonly described as the mitochondrial production of αKG from Glu (via F_23_—Figure 1) (Ahn & Antoniewicz, 2013; Mulukutla et al., 2012; Nicolae et al., 2014). Crucially, the implicit preceding step is Glu transport into mitochondria, which occurs either through the Asp‐Mal shuttle Glu/Asp Aralar1/Aralar2 antiporter (F_20_) or the Asp‐Mal shuttle‐independent GCH1 Glu/H^+^ antiporter (F_30_). Our results indicate an alternative mechanism for Glu anaplerosis, where it is converted, with the consumption of mitochondrial Asp, into αKG via F_22_. This alternative mechanism arises from the high Asn uptake by our GS‐CHO cells, where this nutrient is funneled toward cytosolic malate through reactions $F_{51}$, $F_{31}$, and $F_{24}$. This Asn overflow pushes αKG out of the mitochondrial matrix, where it can be consumed to produce Glu, mainly through $F_{31}$. Irrespective of the metabolic route, the magnitudes of Glu anaplerosis and cataplerosis are consistent with those determined through Metabolic Flux Analysis (Ahn & Antoniewicz, 2013; Nicolae et al., 2014).

#### 3.4.3 Asparagine and aspartate are key anaplerotic nutrients

The asparagine/aspartate pair (Asn/Asp), linked through F_51_, is an important contributor to TCA flux through its sequential conversion to oxaloacetate (F_31_) and malate (F_24_), the latter of which is transported into mitochondria via the Mal/αKG oxoglutarate/malate carrierprotein antiporter (F_21_) for consumption in TCA.

**Figure 5:**
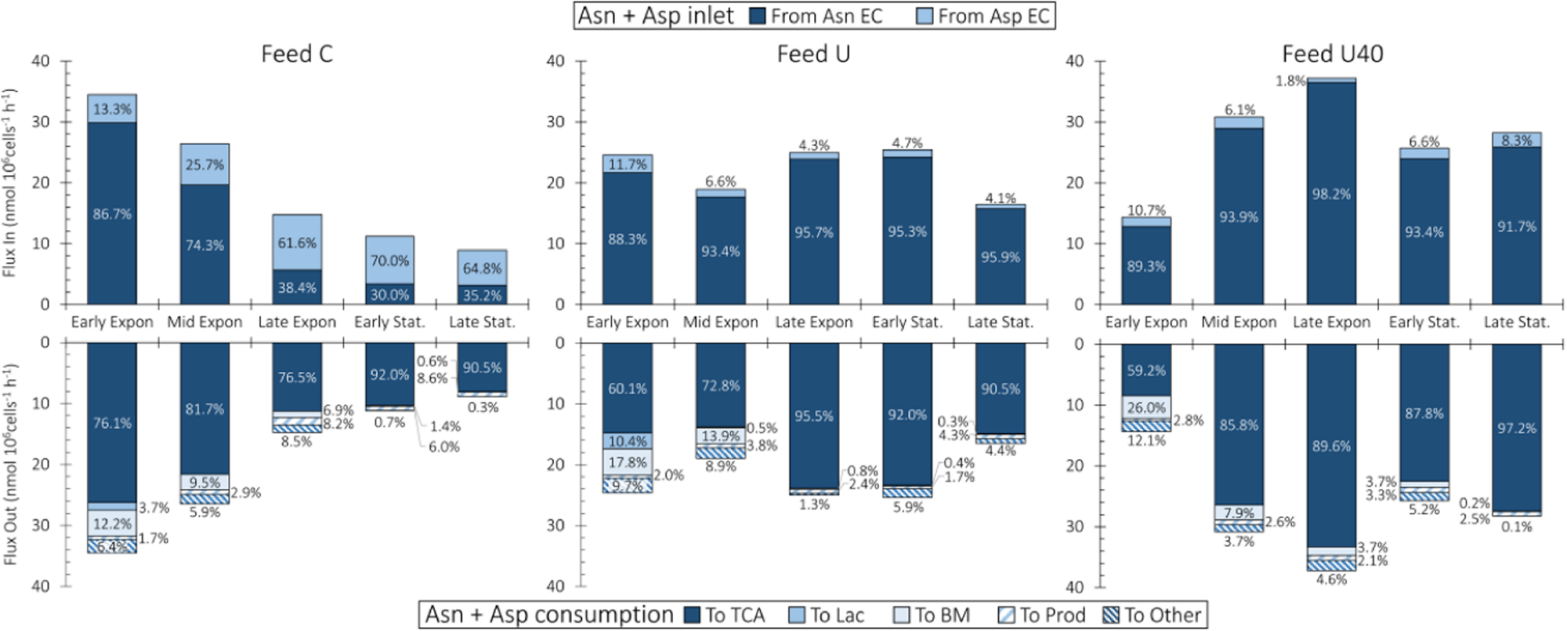
Asparagine + aspartate flux distributions. The sources for asparagine (Asn) and aspartate (Asp) for all feed compositions are shown in the top half and the sinks are shown in the bottom half. The source and sink fluxes for Asn and Asp have been summed for simplicity. The different Asn + Asp sources and sinks are indicated by the shading and the top and bottom legends. The percent contributions to the total sources and sinks are shown within the bars.

During the early exponential interval, cells cultured with Feed C consume 76.1% of Asn/Asp toward TCA (Figure 5—bottom). As culture progresses, the proportion of Asn/Asp channeled toward TCA remains between 76.5% and 81.7% to exceed 90% during the final two intervals. The trend for Asn/Asp consumption toward TCA is even more pronounced for cells cultured with Feeds U and U40, where it exceeds 85% across the final three culture intervals.

Across all feeds, the maximum proportion of Asn/Asp consumed toward biomass and mAb product is 28.8% for Feed U40 during early exponential phase (Figure 5—bottom). These results show that Asn/Asp are fed well beyond stoichiometric requirements for growth and product formation, and that anaplerosis provides an overflow pathway for when these nutrients are fed in excess. These results are consistent with prior work where excess Asn/Asp feeding has been found to increase Ala and Lac secretion by CHO cells (Calmels et al., 2019; Selvarasu et al., 2012).

The anaplerotic overflow pathway for Asn/Asp results in rapid uptake of Asn and a concomitant reduction in its concentration in the culture medium (Supporting Information: Figure 1). The low residual Asn concentrations observed in the Feed C culture, where Asn and Asp are fed at the lowest levels, could be interpreted as being growth limiting. However, our FBA results demonstrate the contrary: increasing Asn feeding was found to slow cell growth (Supporting Information: Figure 1), likely due to excess ammonia production. Similar observations have been reported by Calmels et al. (2019), who performed GeM FBA calculations on industrial CHO DG44 cells.

Our FBA also allows us to estimate the Asn/Asp uptake rates at which no more of this pair can be funneled toward TCA. The raw experimental data shows that Asp accumulates in the extracellular environment of Feed U40 cultures during the late exponential, early stationary, and stationary phases (Supporting Information: Figure 1) implying that, under these conditions, the cells resort to secreting Asp instead of consuming it toward central carbon metabolism. The flux of Asn/Asp toward TCA during these culture phases are 33.3, 22.6, and 27.5 nmol/10^6^ cells/h, respectively, and represent the range of Asn/Asp overflow our GS‐CHO cells can cope with. Interestingly, these flux values correlate closely with those of total Glu production (6.9, 5.5, and 6.1 nmol/10^6^ cells/h for the corresponding culture intervals), indicating that Glu availability may regulate Asn/Asp anaplerosis.

#### 3.4.4 NH_4_^+^ sources and sinks

NH_4_^+^ is a key determinant of CHO cell culture performance because it is known to impact cell growth (Synoground et al., 2021; Wahrheit et al., 2014) and product quality (Borys et al., 1994; Hong et al., 2010). In standard CHO cells, NH_4_^+^ is mainly generated as a by‐product of Gln anaplerosis (glutaminolysis) (Dean & Reddy, 2013; Hong et al., 2010; Wahrheit et al., 2014). Glutamine synthase (GS) cells satisfy their Gln requirements by producing it from Glu via ectopic GS expression. Despite considerable reductions in NH_4_^+^ accumulation, GS‐CHO cells still produce ammonia to levels that may impact product glycosylation (Borys et al., 1994; Hong et al., 2010), so it is therefore important to characterize the major sources and sinks of this key metabolite.

**Figure 6:**
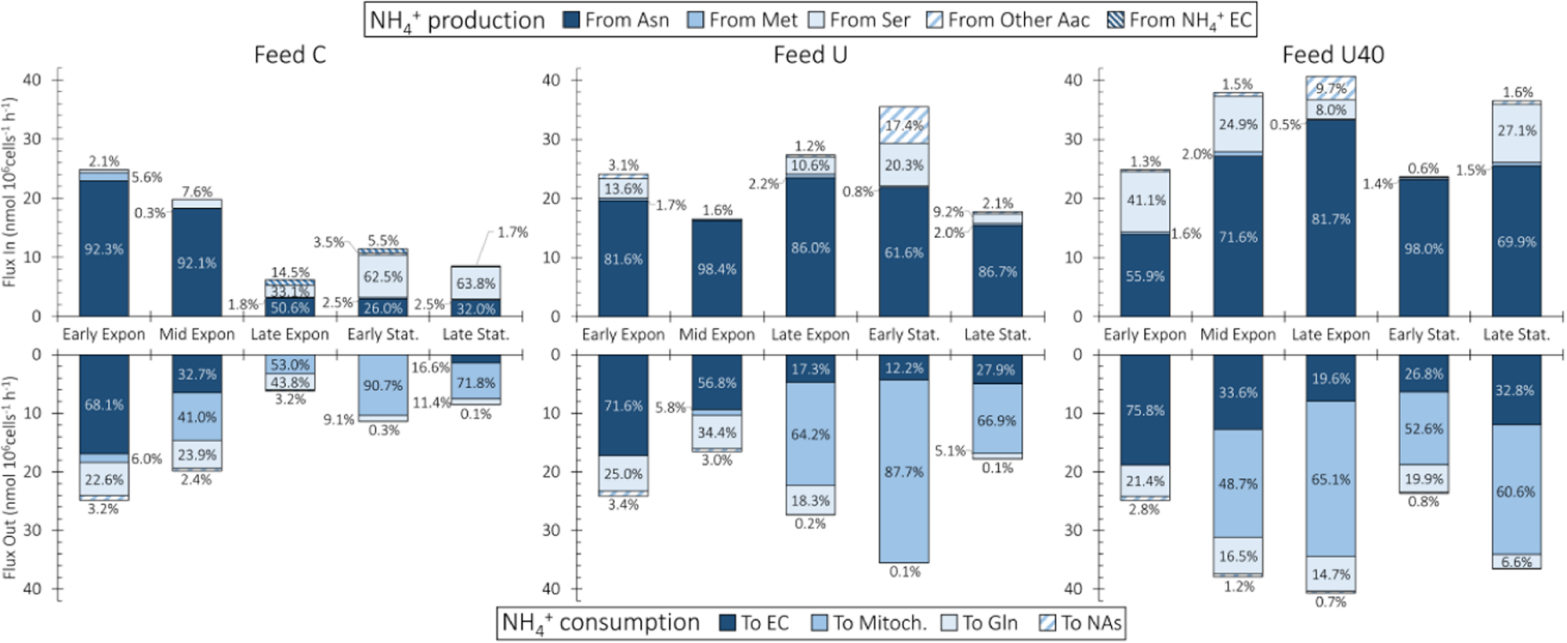
NH_4_^+^ flux distributions. The NH_4_^+^ sources for all feed compositions are shown in the top half and the sinks are shown in the bottom half. The different sources and sinks are indicated by the shading and the top and bottom legends. The percent contribution of each source/sink to the total produced/consumed is shown within the bars.

The dark blue bars in the top half of Figure 6 show that the majority (>50%) of ammonia is produced from asparagine (F_51_). The only exceptions are the Early and Late Stationary intervals of Feed C where, respectively, Asn is the source of 26.0% and 32.0% of all produced NH_4_^+^. During the Early and Late Stationary phases of Feed C, the lower levels of NH_4_^+^ production are due to Asn depletion in the culture media along with NH_4_^+^ uptake by the cells (Supporting Information: Figure 1). An interesting dip in the proportion of NH_4_^+^ produced from Asn is observed in Feed U, where the value drops from 89.0% in Late Exponential to 67.5% during Early Stationary interval to rebound to 86.7% in Late Exponential. This dip is likely caused by glutaminolysis: this is the only interval across all experiments where Gln is consumed by the cells (Supporting Information: Figure 1). Additional sources of NH_4_^+^ include Ser (F_36_), Thr (F_37_), and His (F_54_), although to much lower levels. These results indicate that, in GS‐CHO cells, NH_4_^+^ production is mainly caused by Asn/Asp anaplerosis, which is consistent with previous work with GS‐CHO cells (Calmels et al., 2019; Carinhas et al., 2013).

The total production rate of NH_4_^+^ further confirms its link with Asn/Asp anaplerosis. The top half of Figure 6 shows that the total amount of NH_4_^+^ produced by the cells increases with higher levels of Asn feeding. After the Mid Exponential interval, cells cultured with basal Asn feeding levels (Feed C) have NH_4_^+^ production rates below 11.5 nmol/10^6^ cells/h, whereas the Feed U and Feed U40 cultures (increasingly higher levels of Asn feeding) produce above 20 nmol/10^6^ cells/h of NH_4_^+^.

The bottom half of Figure 6 presents the major NH_4_^+^ sinks across the three feed conditions, where three predominate: (i) secretion to the extracellular environment (dark blue bars, F_108_), (ii) consumption in mitochondria (intermediate blue bars), and (iii) consumption toward Gln synthesis (light blue bars, F_32_). Of the three major sinks, extracellular secretion can be deduced from the experimental data. The sink toward Gln synthesis is also intuitive and occurs because GS‐CHO cells produce Gln from Glu and NH_4_^+^ using ectopically expressed GS.

The mitochondrial NH_4_^+^ sink is the less intuitive one. Our results show that a considerable amount of NH_4_^+^ is consumed by a mitochondrial reaction cycle, where OAA is combined with Glu to produce αKG and Asp (F_22_), and where the resulting αKG is combined with NH_4_^+^ to produce Glu (F_23_). This mitochondrial NH_4_^+^ sink is coupled with the Asp‐Mal shuttle through F_20_, which transports Asp out of mitochondria. If Asp accumulates within mitochondria, F_22_ and F_23_ will be reversed, and net NH_4_^+^ production will occur. These results are consistent with past work where high NH_4_^+^ concentrations increased the uptake rates of Asp and Glu (Lao & Toth, 1997).

Due to the constraint imposed on F_20_ by our FBA solution strategy (Table 1), a second sink for mitochondrial Asp is required to drive mitochondrial NH_4_^+^ consumption. Within CHOmpact, this additional sink is given by F_59_ of the urea cycle, where mitochondrial Asp is irreversibly combined with citrulline to produce fumarate and arginine (Supporting Information: Table 1). Our results indicate that F_59_ consumes over half of the mitochondrial Asp across all culture conditions (Supporting Information: Figure 7) and is, therefore, a key determinant of mitochondrial NH_4_^+^ consumption.

This mitochondrial Asp sink (F_59_) enables NH_4_^+^ consumption that is independent of the Asp‐Mal shuttle and, therefore, of cytosolic Glu availability. This Glu‐independent NH_4_^+^ detoxification pathway requires diverting aKG to F_23_. The produced Glu would then react with TCA‐derived oxaloacetate to replenish aKG and produce mitochondrial Asp (F_22_). Finally, mitochondrial Asp would be consumed by F_59_ to yield arginine and fumarate, which could also feed back into TCA.

CHO cells are reported to produce only trace amounts of urea (Zamorano et al., 2010), indicating that certain enzymes of the cycle may be inactive. The mitochondrial sink identified by CHOmpact may be a urea‐independent, alternate route of NH_4_^+^ detoxification that leverages two urea cycle enzymes (EC 6.3.4.5 and EC 4.3.2.1), which are expressed in CHO cells (Heffner et al., 2020). It is worth noting that both enzymes are known to be expressed at low levels in CHO K1 cells, so their ability to contribute to ammonia detoxification remains to be confirmed.

## 4 CONCLUDING REMARKS

We have presented a compact reaction network to describe the metabolism of mAb‐producing CHO cells. Our reduced metabolic network (144 reactions) performs comparably with the iCHO1766 GeM (>6000 reactions) in predicting the growth rates of different CHO cell lines. Our FBA framework also allowed us to identify the absence of cellular weight and composition measurements as the most likely cause of inaccuracies in predicting growth rates. We have also presented a comprehensive optimization strategy that constrains the solution space to yield physiologically consistent flux distributions across all phases of cell culture.

When coupled with nonlinear optimization, CHOmpact greatly enhances the interpretability of metabolic flux distributions across different phases of cell culture. Our results provide insights into the mechanisms underlying Glu anaplerosis and its dependence on the uptake of Asn/Asp. We have also identified Asn/Asp as the key anaplerotic nutrients of GS‐CHO cells, where they act as a considerable source of lactate during the early stages of culture.

Our results also show that Asn is the predominant source of NH_4_^+^ across all culture conditions and that a major sink for this key metabolite is consumption within mitochondria. The presence of Asp within mitochondria determines whether this organelle is a source or sink of NH_4_^+^: when Asp accumulates, mitochondria can become a net source of NH_4_^+^, when Asp is depleted, NH_4_^+^ is consumed within mitochondria. The Asp‐Mal shuttle determines the intracellular flux distributions of Asn, Asp, Gln, Glu, and NH_4_^+^. Our FBA solution strategy constrains fluxes through the Asp‐Mal to not exceed the flux of “free” Glu entering or produced by the cells to obtain physiologically consistent flux distributions for Asn, Asp, Gln, Glu, and NH_4_^+^.

Moving forward, the enhanced understanding of metabolic dynamics afforded by our CHOmpact reaction network and nonlinear optimization framework can be used to define feeding strategies that optimize cell culture performance. Furthermore, the compact size of our reaction network will also facilitate the creation of hybrid dynamic FBA/culture dynamics models, which can be used as digital twins for dynamic optimization and control of cell culture bioprocesses.

## AUTHOR CONTRIBUTIONS

**Ioscani Jimenez del Val**: Conceptualisation, methodology, investigation, resources, data curation, writing—original draft, review and editing, visualisation, funding acquisition. **Sarantos Kyriakopoulos**: Investigation, data curation. **Simone Albrecht**: Investigation, data curation. **Henning Stöckmann**: Investigation, data curation. **Pauline M. Rudd**: Resources, supervision, writing—review and editing. **Karen M. Polizzi**: Supervision, resources, funding acquisition. **Cleo Kontoravdi**: Supervision, resources, writing—review and editing, funding acquisition.

## CONFLICT OF INTEREST STATEMENT

The authors declare no conflict of interest.

## Supporting information

Supporting information.

Supporting information.

## Data Availability

The data that support the findings of this study are available from the corresponding author upon reasonable request.
