# Supplementary material for: CHOmpact: A reduced metabolic model of Chinese hamster ovary cells with enhanced interpretability
Source: Biotechnol Bioeng. 2023 Jun 5;120(9):2479–93. doi: 10.1002/bit.28459 (PMC10952303; doi:10.1002/bit.28459)
Supplement: Supplementary file 1 — Supporting information. [file BIT-120-2479-s002.pdf]

Supplementary File

CHOmpact: a reduced metabolic model of Chinese hamster ovary cells with enhanced interpretability

Ioscani Jiménez del Val[1,*], Sarantos Kyriakopoulos[2,‡], Simone Albrecht[3], Henning Stockmann[3], Pauline M Rudd[3,†], Karen M Polizzi[4] and Cleo Kontoravdi[4]

[1] School of Chemical & Bioprocess Engineering, University College Dublin D04 V1W8, Ireland
[2] MS&T, BioMarin Manufacturing Ireland, Cork P43 R298, Ireland
[3] NIBRT GlycoScience Group, National Institute for Bioprocessing Research and Training, Dublin A94 X099, Ireland
[4] Department of Chemical Engineering, Imperial College London SW7 2AZ, United Kingdom
*Author to whom correspondence should be addressed.
e-mail: ioscani.jimenezdelval@ucd.ie

† Present address: Bioprocessing Technology Institute, Agency for Science, Technology and Research (A*STAR) 138668, Singapore
‡ Present address: Drug Product Development, Janssen Pharmaceuticals, 8200 Schaffhausen, Switzerland

Aspartate-Malate Shuttle

The purpose of the Asp-Mal shuttle is to transfer reducing equivalents (electrons) from cytosolic NADH to mitochondrial NAD^+ (Borst, 2020; Zagari et al., 2013). Because the mitochondrial membrane is impermeable to electrons, the following reactions are required for their transfer:

(+1) F_{15}	$Mal_{mit} + NAD^+_{mit}$	$\leftrightarrow$	$Oxal_{mit} + NADH_{mit}$
(+1) F_{20}	$Glu_{cyt} + Asp_{mit}$	$\rightarrow$	$Glu_{mit} + Asp_{cyt}$
(+1) F_{21}	$Mal_{cyt} + \alpha KG_{mit}$	$\leftrightarrow$	$Mal_{mit} + \alpha KG_{cyt}$
(+1) F_{22}	$Oxal_{mit} + Glu_{mit}$	$\leftrightarrow$	$\alpha KG_{mit} + Asp_{mit}$
(+1) F_{24}	$Oxal_{cyt} + NADH_{cyt}$	$\leftrightarrow$	$Mal_{cyt} + NAD^+_{cyt}$
(+1) F_{31}	$Asp_{cyt} + \alpha KG_{cyt}$	$\leftrightarrow$	$Oxal_{cyt} + Glu_{cyt}$
Sum	$NADH_{cyt} + NAD^+_{mit}$	$\rightarrow$	$NADH_{mit} + NAD^+_{cyt}$

From the above, it can be seen that the Asp-Mal shuttle reactions yield no net production/consumption of Asp, Glu, αKG, Oxal or Mal because, within this pathway, all participating components (except $NADH_{cyt}$, NAD^+_{mit}, $NADH_{mit}$, and NAD^+_{cyt}) are produced and consumed at equivalent rates – their fluxes within the shuttle cancel out. In conjunction, the sink for mitochondrial $NADH_{mit}$ is unconstrained: it is consumed to produce ATP via F_{19} and the flux of ATP consumption towards unspecified reactions (F_{104} – see Figure 1 in the manuscript) is computed through optimisation.

Overall, this means that as long as the balances for $NADH_{cyt}$, NAD^+_{mit}, $NADH_{mit}$, and NAD^+_{cyt} are satisfied, the fluxes for all reactions in the Asp-Mal shuttle can take any value and may lead to shuttle uptakes of glutamate that are unfeasibly high (e.g., having similar magnitude as glycolytic fluxes while glutamate uptake from the extracellular environment is an order of magnitude below that of glucose).

For this reason, we have constrained F_{20}, the rate-limiting step of the Asp-Mal shuttle (LaNoue, Meijer, & Brouwer, 1974; LaNoue & Tischler, 1974), to never exceed the flux of glutamate internalised by the cells or produced through reactions independent of the Asp-Mal shuttle.

Cell culture data

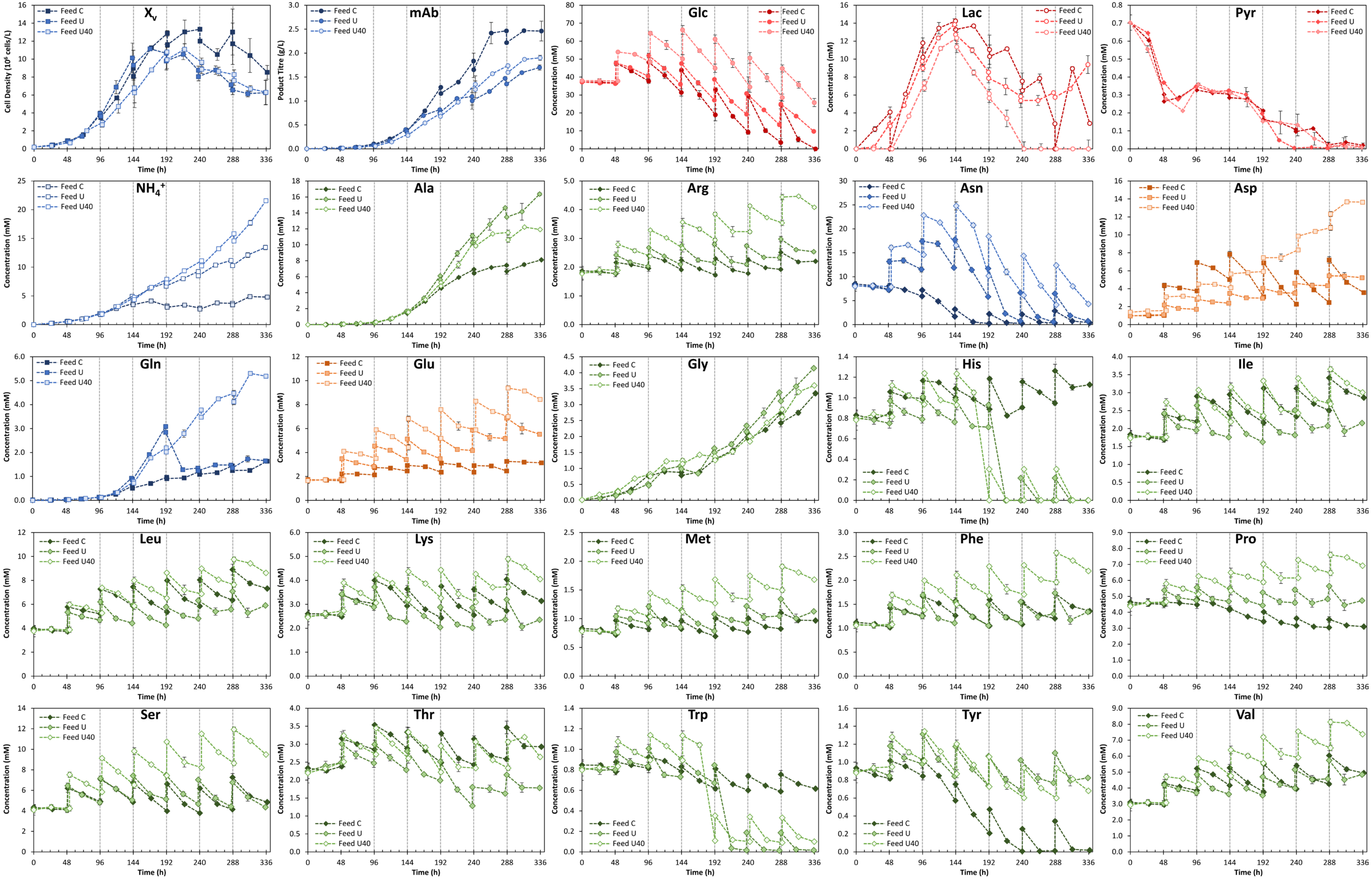

Supplementary Figure 1. Experimental data for three fed-batch experiments. The vertical grey dashed lines show the culture intervals considered for FBA calculations.

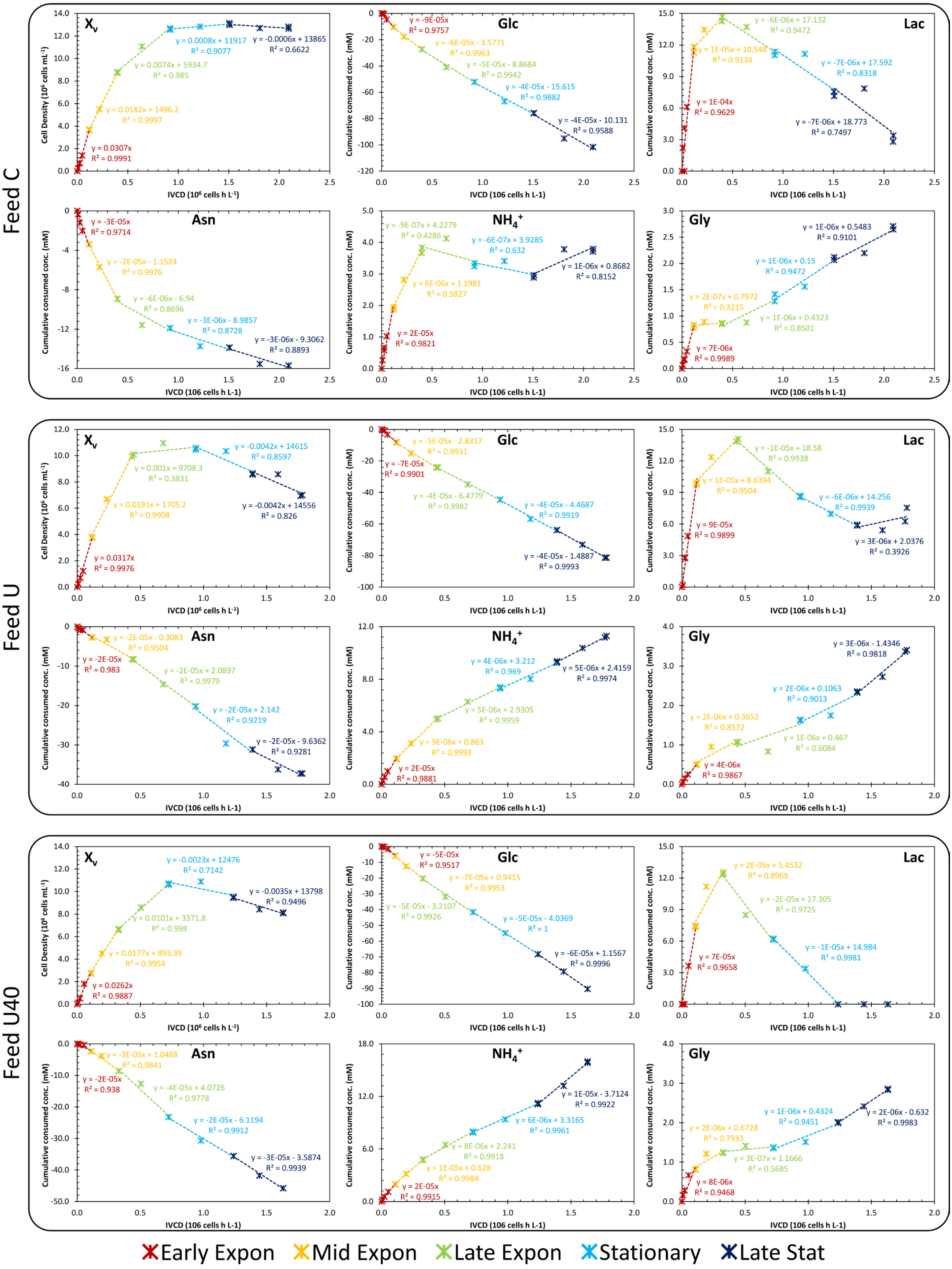

Supplementary Figure 2. Intervals considered for flux analysis

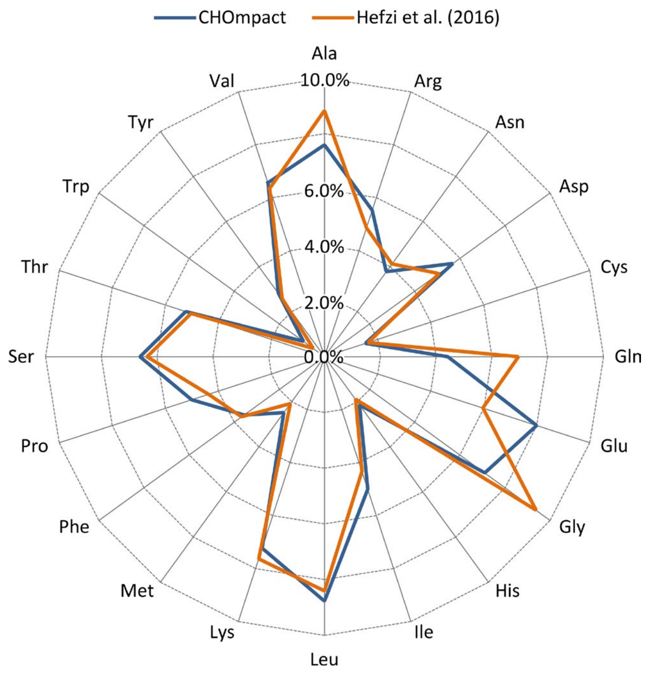

Supplementary Figure 3. Comparison of the amino acid composition in HCPs.
The values considered in this paper are shown in blue and the ones assumed by Hefzi et al. (2016) are presented in orange. The composition is shown in units of mole of amino acid *i* per mole of host cell protein.

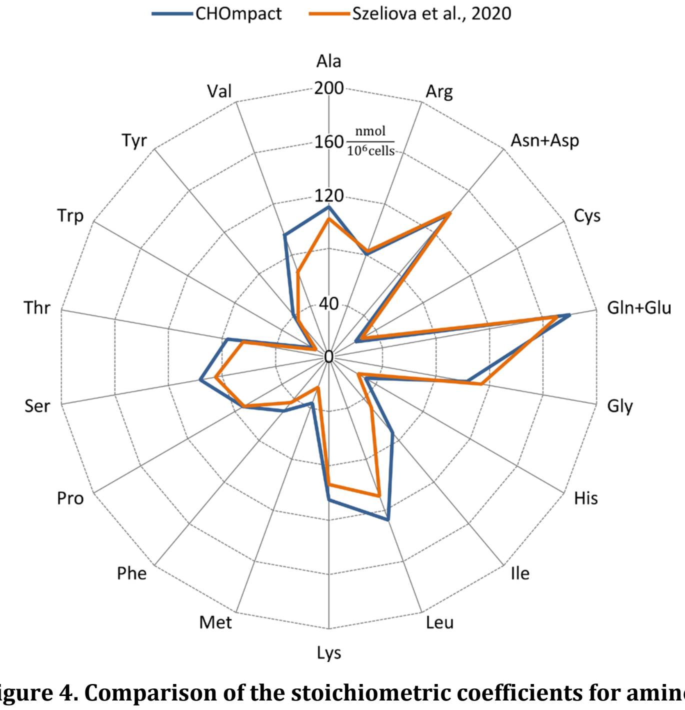

Supplementary Figure 4. Comparison of the stoichiometric coefficients for amino acids in biomass.
The exponential growth stoichiometric coefficients of amino acids in biomass used in this work are compared with those determined by Szeliova et al. (2020). The latter correspond to the average value across ten CHO cell lines cultured under different nutrient conditions.

CHOmpact Objective Function analysis: initial values for decision variables & local minima

The CHOmpact objective function is non-linear. Because of this, there is uncertainty on which local optimum is being obtained and whether the value is indeed the optimal one across the solution space. The solution obtained through non-linear optimisation is particularly contingent on the initial values (guesses) for the decision variables. To solve all optimisation problems in the present work, the nonlinear programming sequential quadratic programming solver (NLPSQP) built into gPROMS ModelBuilder v7.0 was used. Specifically, the gPROMS's NLPSQP solver uses four tolerances (Feasibility Tolr, which sums all constraint and bound violations; Complementarity Tolr, which weighs contraint violations by their influence on the gradient of the OF; Taylor Tolr, which represents the rate of change in the objective function value between iterations; Stationarity Tolr, which measures the proximity to a local optimum) to obtain local optima for a given problem (Process Systems Enterprise, 2021).

To assess the consistency and robustness with which the optimum point is found, we systematically varied the initial values of four key optimisation decision variables, F_{77}, F_{103}, F_{104}, and F_{106}. All other optimisation decision variables were excluded from this analysis because they correspond to the values of transport fluxes to/from the cells whose initial guesses were always set to experimentally determined values.

Specifically, 81 optimisations were run with combinations of three initial guesses – low, medium, and high – for each of the four decision variables ($3^4 = 81$) using input data for the Feed C Early Exp culture interval. This culture interval was selected because it was observed to have the highest objective function value and the highest optimisation failure rate (by failing to meet the four tolerance critera outlined above) across all optimisations. The four decision variables and their tested initial values for optimisation were:

				(nmol/10⁶cells/h)		
**Flux**	**Reaction**	**Enzyme ID**	**Accession No.**	**L**	**M**	**H**
F_{77}	$NADP^+ \rightarrow NAD^+$	3.1.3.1	XP_003510743	1.0	125	249
F_{103}	$NADH_{cyt} \rightarrow NAD^+$	1.6.2.2	XP_003498894	1.0	125	249
F_{104}	ATP → ADP	Maintenance	Multiple	10.0	1250	2490
F_{106}	$CO_2 \rightarrow CO_{2,\,EC}$	Excretion	Multiple	5.0	500	995

Results for the 81 optimisations are presented in Supplementary Figure 4. Of the 81 optimisations, 25 failed because they did not meet the Complementarity (set at 1E-12) and/or the Taylor (set at 1E-12) tolerances. Of the 56 optimisations that successfully converged, thirty reached the lowest objective function value of 552.28, eighteen obtained an OF value between [552.29, 586.12], and eight reached a considerably higher OF value of between [713.64, 810.52] (orange dataset in Supplementary Figure 4). This suggests that three local minima are present across the feasible solution range, of which the lowest one is found with over half the combinations of decision variable initial guesses.

When analysing the contribution of each OF term to the overall OF value (blue datasets in Supplementary Figure 4) – computed as the difference between the OF term value minus the median value (ΔOF), the main deviations arise from the by-product (BP) term ($\sum F_{k,BP}^2$) with only minor contributions from the SSE term. This data suggests that, for a small subset of initial guesses for the decision variables, the NLPSQP solver lands in a local minimum – associated with the BP OF term – and continues minimising the other terms.

Although the data indicates that the solutions obtained do depend on the initial values for decision variables, the majority (>50%) of the initial value sets reach the minimum objective function value. In addition, our analysis has also identified the subset of initial values that are more likely to reach OF minima. In all optimisations presented in the manuscript, at least three sets of initial values (from across the successful subset) were run to ensure that the OF minimum was obtained.

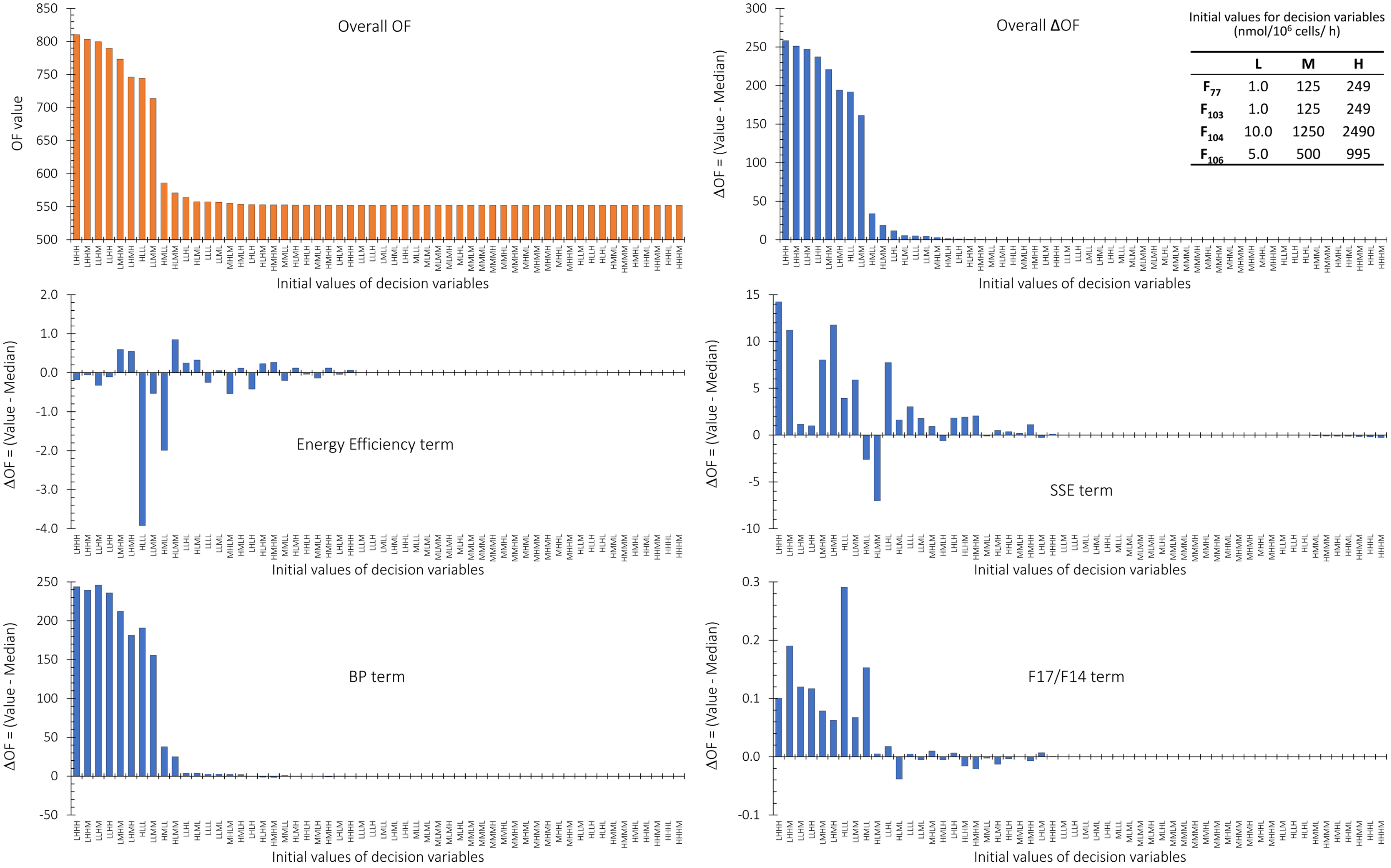

	L	M	H
F_{77}	1.0	125	249
F_{103}	1.0	125	249
F_{104}	10.0	1250	2490
F_{106}	5.0	500	995

Supplementary Figure 5. Objective function (OF) values obtained with different sets of initial values for optimisation decision variables.Top left shows the overall OF value with different sets of initial guesses for decision variables. Top right presents the difference between the OF value and the median OF value obtained across all 81 optimisations. The remaining graphs show the contribution of each OF term to the overall OF value (presented as the OF term value minus the median OF term value across the 81 optimisations).

CHOmpact Objective Function performance vs. MIN(SSE)

Performance of the CHOmpact objective function (OF) was compared with sum of square error minimisation (SSE), which is commonly done in standard metabolic flux analysis. The top panel of Supplementary Figure 3 presents the difference in magnitude among central carbon metabolic fluxes by subtracting the flux values obtained with the CHOmpact OF from those obtained through SSE minimisation: $\Delta F_k = F_{k,SSE} - F_{k,CHOmpact}$. The bottom panel shows the relative difference in computed fluxes as obtained with the following equation:

$$\Delta F_k = \frac{F_{k,SSE} - F_{k,CHOmpact}}{MAX(F_{k,SSE}, F_{k,CHOmpact})}$$

Considerable differences can be seen in the aspartate/malate shuttle fluxes (F_{20} to F_{24}), where SSE obtains much higher flux values for all three feeds, especially those associated with the aspartate/malate shuttle. To highlight potential biological inconsistencies, the SSE optimisation computes an F_{20} (the Asp/Mal shuttle transport of Glu into mitochondria) flux of at least 60 nmol/10^6 cells/h across the Mid Expon., Late Expon., and Early Stat. intervals for Feed C, whereas a maximum value of 6.9 nmol/10^6 cells/h is obtained with the CHOmpact OF. For those intervals, Glu uptake from the culture media does not exceed 1.3 nmol/10^6 cells/h. It would seem unlikely that the cells to produce Glu at such high rates. Potential inconsistencies in the Asp/Mal shuttle fluxes calculated with the SSE OF arise from the shuttle's cyclical nature, as discussed on page 1, above. The CHOmpact OF has been formulated specifically to address such concerns and yields more biologically consistent intracellular flux distributions.

	Feed C					Feed U					Feed U40				
	Early Expon.	Mid Expon.	Late Expon.	Early Stat.	Late Stat.	Early Expon.	Mid Expon.	Late Expon.	Early Stat.	Late Stat.	Early Expon.	Mid Expon.	Late Expon.	Early Stat.	Late Stat.
ΔF_9	18.88	-4.18	0.77	1.86	-6.13	2.73	1.02	-6.70	-3.27	5.60	-1.50	-3.80	0.42	-3.17	-3.45
ΔF_{10}	19.29	-4.31	0.50	1.72	-6.40	2.64	0.87	-6.93	-3.36	5.83	-1.56	-3.93	0.56	-3.33	-3.53
ΔF_{11}	20.32	-4.31	0.59	1.77	-6.38	4.28	0.86	-6.90	-2.25	5.84	-1.57	-3.50	1.47	-3.38	-3.48
ΔF_{12}	18.64	-2.18	1.07	3.67	-4.53	3.83	1.09	-3.66	1.19	6.22	-1.58	-1.83	2.04	-2.42	-1.44
ΔF_{13}	20.62	-2.88	0.62	3.35	-5.53	4.03	0.73	-4.57	0.34	6.48	-1.57	-2.47	2.24	-3.09	-2.09
ΔF_{14}	26.27	-12.76	0.89	-1.42	-9.06	4.86	-3.31	-11.34	0.13	5.87	-3.98	-9.51	0.08	-8.90	-6.68
ΔF_{15}	29.34	44.97	70.03	45.47	-18.59	7.97	33.22	-39.31	-1.51	5.19	-18.40	-12.37	-0.88	-24.96	-16.56
ΔF_{16}	-0.87	-0.60	0.00	0.00	0.00	-0.68	0.00	0.00	3.04	0.00	3.54	4.62	-0.01	3.67	0.00
ΔF_{17}	4.15	-3.90	-2.84	1.25	0.16	1.88	-3.02	-1.51	9.53	0.86	3.46	6.17	1.69	-1.74	1.43
ΔF_{18}	23.86	-3.32	0.71	3.49	-6.25	3.73	0.53	-5.02	-0.51	7.62	-1.58	-3.05	2.63	-3.41	-2.27
ΔF_{19}	85.73	34.79	66.17	54.83	-33.43	16.52	30.81	-54.73	7.61	20.41	-19.34	-8.70	3.46	-36.06	-19.85
ΔF_{20}	4.94	58.43	69.00	48.41	-8.92	4.00	36.26	-25.83	5.04	0.19	-10.93	3.15	0.90	-12.32	-8.53
ΔF_{21}	7.22	53.82	66.30	48.14	-9.37	5.00	33.51	-29.49	7.89	0.18	-10.96	3.31	0.72	-17.80	-8.46
ΔF_{22}	9.60	48.56	69.26	43.61	-12.46	4.57	32.21	-32.61	4.80	-0.41	-13.36	-3.95	-1.30	-18.12	-13.12
ΔF_{23}	1.35	-5.27	2.87	-4.59	-3.11	-2.07	-1.29	-3.15	-4.19	-0.60	-2.40	-7.68	-2.94	-0.27	-4.72
ΔF_{24}	7.07	53.89	66.40	48.42	-8.92	5.16	33.55	-29.31	8.26	0.25	-10.96	3.31	0.71	-17.69	-8.46
ΔF_{29}	-1.44	4.53	2.61	0.00	0.00	-0.88	2.70	3.47	-2.88	-0.06	0.00	0.00	0.75	5.33	0.00
ΔF_{30}	3.31	-4.61	-2.61	-0.22	-0.42	2.64	-2.76	-3.62	3.95	-0.01	-0.02	0.58	0.74	-5.53	0.13
ΔF_{31}	5.63	58.42	69.01	48.42	-8.92	4.27	36.25	-25.84	5.37	0.19	-10.96	3.31	1.46	-12.36	-8.46

	Feed C					Feed U					Feed U40				
	Early Expon.	Mid Expon.	Late Expon.	Early Stat.	Late Stat.	Early Expon.	Mid Expon.	Late Expon.	Early Stat.	Late Stat.	Early Expon.	Mid Expon.	Late Expon.	Early Stat.	Late Stat.
ΔF_9	17.6%	-3.6%	0.7%	1.6%	-5.4%	4.6%	1.2%	-5.4%	-2.4%	5.1%	-3.2%	-2.6%	0.3%	-2.5%	-2.2%
ΔF_{10}	18.0%	-3.7%	0.5%	1.5%	-5.7%	4.5%	1.0%	-5.6%	-2.5%	5.3%	-3.4%	-2.7%	0.4%	-2.6%	-2.2%
ΔF_{11}	19.9%	-3.8%	0.6%	1.5%	-5.7%	7.2%	1.0%	-5.5%	-1.6%	5.2%	-3.3%	-2.3%	1.0%	-2.7%	-2.1%
ΔF_{12}	18.5%	-2.0%	1.0%	3.2%	-4.0%	6.3%	1.3%	-2.9%	0.8%	5.5%	-3.1%	-1.2%	1.3%	-1.9%	-0.9%
ΔF_{13}	19.4%	-2.6%	0.6%	2.8%	-4.8%	6.5%	0.9%	-3.6%	0.2%	5.7%	-3.0%	-1.6%	1.4%	-2.4%	-1.3%
ΔF_{14}	19.5%	-9.2%	0.8%	-1.0%	-7.0%	6.5%	-3.5%	-6.6%	0.1%	4.2%	-6.5%	-4.6%	0.0%	-5.6%	-3.0%
ΔF_{15}	17.8%	21.4%	37.3%	23.7%	-13.6%	8.4%	22.7%	-20.4%	-0.7%	3.4%	-25.3%	-5.4%	-0.4%	-14.1%	-6.8%
ΔF_{16}	-100.0%	-100.0%	-0.8%	0.2%	0.7%	-100.0%	0.1%	0.0%	87.5%	0.3%	88.0%	87.6%	-1.0%	87.7%	-0.2%
ΔF_{17}	14.0%	-20.2%	-60.8%	7.6%	1.6%	9.5%	-22.6%	-5.3%	24.6%	4.9%	17.0%	14.4%	5.3%	-9.2%	3.9%
ΔF_{18}	20.8%	-2.8%	0.7%	2.8%	-5.3%	5.8%	0.6%	-3.7%	-0.3%	6.5%	-2.6%	-1.8%	1.6%	-2.6%	-1.3%
ΔF_{19}	16.8%	6.2%	13.4%	10.4%	-7.1%	5.4%	7.5%	-9.7%	1.2%	4.3%	-8.3%	-1.3%	0.5%	-6.4%	-2.7%
ΔF_{20}	15.1%	71.1%	91.4%	78.4%	-93.7%	15.3%	67.8%	-100.0%	17.2%	1.2%	-55.0%	8.2%	2.8%	-62.2%	-26.9%
ΔF_{21}	12.0%	54.3%	84.8%	67.0%	-53.3%	12.4%	52.4%	-59.4%	16.5%	0.6%	-38.6%	5.1%	1.2%	-47.5%	-14.3%
ΔF_{22}	16.6%	49.8%	84.7%	55.0%	-52.2%	12.6%	53.1%	-48.1%	5.7%	-1.0%	-49.9%	-4.8%	-1.5%	-37.2%	-15.5%
ΔF_{23}	47.3%	-64.7%	46.6%	-44.3%	-50.7%	61.3%	-135.2%	-18.0%	-13.4%	-5.0%	66.2%	-41.7%	-11.1%	-2.2%	-21.3%
ΔF_{24}	11.7%	54.1%	84.6%	67.2%	-50.8%	12.7%	52.1%	-58.7%	17.1%	0.9%	-38.6%	5.1%	1.2%	-46.8%	-14.3%
ΔF_{29}	-65.1%	97.9%	28.7%	100.0%	0.0%	-20.8%	77.9%	100.0%	-31.8%	-1.9%	28.7%	0.0%	9.4%	49.3%	0.0%
ΔF_{30}	14.8%	-27.1%	-97.6%	-1.8%	-5.2%	17.0%	-26.9%	-14.8%	13.9%	-0.1%	-0.2%	2.0%	2.7%	-33.7%	0.4%
ΔF_{31}	9.2%	56.1%	78.8%	67.2%	-50.8%	9.7%	53.4%	-51.8%	9.9%	0.6%	-38.6%	5.1%	2.2%	-28.6%	-14.3%

Supplementary Figure 6. Comparison of the CHOmpact OF with SSE minimisation. The top panel presents the magnitude of the difference between fluxes computed through SSE minimisation and those obtained with the CHOmpact objective function. The bottom panel presents the relative differences (%) between the fluxes computed with each OF.

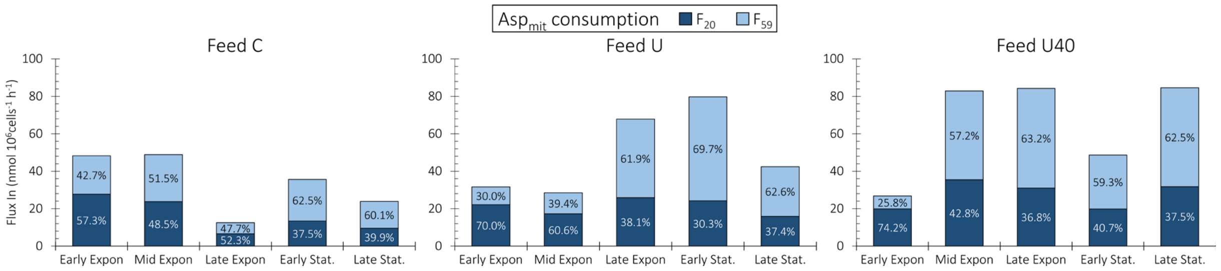

Supplementary Figure 7. The rate-limiting step of the Asp/Mal shuttle (F_{20}) and the sum of cell-specific uptake rates for Asn, Asp, Gln, and Glu for the three feed compositions (Feed C, U, and U40). Similarities between the profiles for F_{20} and the sum of anaplerotic amino acid uptake rates are observed. The minimum values of F_{20} correspond to a shift from Glu cataplerosis to anaplerosis.

References

Borst, P. (2020). The malate-aspartate shuttle (Borst cycle): How it started and developed into a major metabolic pathway. *IUBMB Life, 72*(11), 2241-2259. doi:10.1002/iub.2367

Hefzi, H., Ang, K. S., Hanscho, M., Bordbar, A., Ruckerbauer, D., Lakshmanan, M., . . . Lewis, N. E. (2016). A Consensus Genome-scale Reconstruction of Chinese Hamster Ovary Cell Metabolism. *Cell Syst, 3*(5), 434-443 e438. doi:10.1016/j.cels.2016.10.020

LaNoue, K. F., Meijer, A. J., & Brouwer, A. (1974). Evidence for electrogenic aspartate transport in rat liver mitochondria. *Arch Biochem Biophys, 161*(2), 544-550.

LaNoue, K. F., & Tischler, M. E. (1974). Electrogenic characteristics of the mitochondrial glutamate-aspartate antiporter. *J Biol Chem, 249*(23), 7522-7528.

Process Systems Enterprise. (2021). gPROMS. www.psenterprise.com/products/gproms, 1997-2021.

Szeliova, D., Ruckerbauer, D. E., Galleguillos, S. N., Petersen, L. B., Natter, K., Hanscho, M., . . . Zanghellini, J. (2020). What CHO is made of: Variations in the biomass composition of Chinese hamster ovary cell lines. *Metab Eng*. doi:10.1016/j.ymben.2020.06.002

Zagari, F., Stettler, M., Baldi, L., Broly, H., Wurm, F. M., & Jordan, M. (2013). High expression of the aspartate–glutamate carrier Aralar1 favors lactate consumption in CHO cell culture. *Pharmaceutical Bioprocessing, 1*(1), 19-27. doi:doi:10.4155/pbp.13.5
